# Transcriptome Analysis of Duck and Chicken Brains Infected with Aquatic Bird Bornavirus-1 (ABBV-1)

**DOI:** 10.3390/v14102211

**Published:** 2022-10-08

**Authors:** Phuc H. Pham, Teodora Tockovska, Alexander Leacy, Melanie Iverson, Nicole Ricker, Leonardo Susta

**Affiliations:** Department of Pathobiology, Ontario Veterinary College, University of Guelph, Guelph, ON N1G 2W1, Canada

**Keywords:** aquatic bird birnavirus-1, bornaviruses, transcriptomics, GO, KEGG, lncRNA

## Abstract

Aquatic bird bornavirus 1 (ABBV-1) is a neurotropic virus that infects waterfowls, resulting in persistent infection. Experimental infection showed that both Muscovy ducks and chickens support persistent ABBV-1 infection in the central nervous system (CNS), up to 12 weeks post-infection (wpi), without the development of clinical disease. The aim of the present study was to describe the transcriptomic profiles in the brains of experimentally infected Muscovy ducks and chickens infected with ABBV-1 at 4 and 12 wpi. Transcribed RNA was sequenced by next-generation sequencing and analyzed by principal component analysis (PCA) and differential gene expression. The functional annotation of differentially expressed genes was evaluated by gene ontology (GO) and Kyoto Encyclopedia of Genes and Genomes (KEGG) analysis. The PCA showed that the infected ducks sampled at both 4 and 12 wpi clustered separately from the controls, while only the samples from the chickens at 12 wpi, but not at 4 wpi, formed a separate cluster. In the ducks, more genes were differentially expressed at 4 wpi than 12 wpi, and the majority of the highly differentially expressed genes (DEG) were upregulated. On the other hand, the infected chickens had fewer DEGs at 4 wpi than at 12 wpi, and the majority of those with high numbers of DEGs were downregulated at 4 wpi and upregulated at 12 wpi. The functional annotation showed that the most enriched GO terms were immune-associated in both species; however, the terms associated with the innate immune response were predominantly enriched in the ducks, whereas the chickens had enrichment of both the innate and adaptive immune response. Immune-associated pathways were also enriched according to the KEGG pathway analysis in both species. Overall, the transcriptomic analysis of the duck and chicken brains showed that the main biological responses to ABBV-1 infection were immune-associated and corresponded with the levels of inflammation in the CNS.

## 1. Introduction

Bornaviruses are enveloped viruses with a negative-sense single-stranded RNA genome, which are classified in the *Bornaviridae* family, *Orthobornavirus* genus [1]. The RNA genome of these viruses encodes at least six proteins, which are, from the 3′ to 5′ genomic ends, nucleoprotein (N), phosphoprotein (P), non-structural protein (X), matrix protein (M), glycoprotein (G), and RNA-dependent RNA polymerase (L) [2]. Bornaviruses are characterized by nuclear replication, the establishment of persistent infection (non-lytic cycle), and marked neurotropism in infected species, leading to chronic inflammation of the central and peripheral nervous system, resulting in neurological signs [3].

In mammalian species, Borna disease virus 1 (BoDV-1, the type-species of the *Orthobornavirus* genus, causes Borna disease, a neurological affliction of sheep and horses in central Europe [4]. Aquatic bird bornavirus-1 (ABBV-1), in the *Orthobornavirus avisaquaticae* species, has been identified in wild waterfowl in North America and Europe, and it has been associated with both asymptomatic infections and symptomatic infections (neurological signs, proventricular dilation, and wasting) [5,6,7,8,9,10]. ABBV-1 has a broad host range, and natural infection with this virus has been identified in ratites, raptors, and gulls, which are taxonomically distant from waterfowl species [11,12,13]. Given the circulation of bornaviruses in multiple avian species [14], it is possible that ABBV-1 could spill over and infect domestic poultry. Two recent studies from our group support this notion, as we have shown that ABBV-1 can infect and establish persistent infection in the nervous tissues of Muscovy ducks (*Cairina moschata*) and chickens (*Gallus gallus*), upon intracranial and intramuscular inoculation [15,16]. In these studies, the ducks appeared to be markedly susceptible to infection, as suggested by the high levels of virus replication in their nervous tissues and the widespread virus distribution in multiple visceral organs, as opposed to the chickens, in which the infection was mostly limited to the nervous tissue without visceral involvement, with comparatively low levels of virus replication [15,16]. This partly agrees with another in vitro study, in which we showed that ABBV-1 can readily infect primary and immortalized duck-embryo fibroblasts, but not chicken-embryo fibroblasts [17].

Little is known about the molecular pathogenesis and cellular response to bornavirus infection. To our knowledge, only four microarray studies, two proteomic studies, and one next-generation RNA-sequencing study have been conducted on tissues or cells infected with BoDV-1 [18,19,20,21,22,23,24]. However, no transcriptomic studies have been conducted on avian species infected with bornaviruses in general, or ABBV-1 in particular. Building on our previous experimental infection studies, the goal of this study was to investigate the transcriptomic profiles of the brains of Muscovy ducks and chickens infected with ABBV-1, and to outline a framework of the gene networks associated with ABBV-1 infection in two avian hosts that showed different susceptibility to ABBV-1 infection.

## 2. Materials and Methods

### 2.1. Infection of Ducks and Chickens with ABBV-1 and Extraction of RNA from Collected Samples

This work is an extension of two previously described experimental infection trials [15,16], from which the brain samples were derived. Detailed experimental design and infection protocols for both ducks and chickens can be found in the referenced studies. Relevant methods for interpretation of the present research are briefly reported below.

Groups of day-old Muscovy ducks (*Cairina moschata*) and White Leghorn chickens (*Gallus gallus*) were infected intracranially with 4.5 × 10^5^ focus-forming units (FFUs)/duck or 6.6 × 10^4^ FFUs/chicken. Control birds were inoculated with carrier only. At 4- and 12-weeks post infection (wpi), 7 birds from the infected and control groups were euthanized and brains collected for histopathology, quantification of virus RNA copies, and transcriptome analysis (total, n = 28 per species). Tissues for histopathology were fixed in 10% neutral buffered formalin and processed routinely. The severity of inflammation was scored by assessing the extent and magnitude of perivascular lymphocytic cuffs around vessels, and ranged between 0 and 6, as detailed elsewhere [15,16]. The remainder of the brain tissue was stored in RNA preserving solution (20 mM ethylenediaminetetraacetic acid [EDTA], 25 mM sodium citrate, and 70% (*w*/*v*) ammonium sulfate with a pH of 5.2). The magnitude of virus replication was assessed by RT-qPCR for the ABBV-1 N gene by interpolating the cycle threshold value of the samples against a known dilution of the target region (gene block) and reported as viral RNA copies per 150 ng of total tissue RNA [15,16]. The rest of the tissues were used for the RNAseq experiment.

### 2.2. Library Preparation and RNA Sequencing

Total RNA was extracted from 28 duck and 28 chicken brains using approximately 300 mg of tissues and the E.Z.N.A. RNA Kit II (Omega Bio-Tek, Norcross, GA, USA), according to the manufacturer’s instructions. Preparation of cDNA library and RNA-sequencing work were completed by Genome Quebec (Montreal, QC, Canada). Total RNA quantity and integrity was assessed using a NanoDrop Spectrophotometer ND-1000 (NanoDrop Technologies, Inc., Wilmington, DE, USA) and on a 2100 Bioanalyzer (Agilent Technologies, Santa Clara, CA, USA), respectively. The NEBNext Ultra II DNA Library Prep Kit from Illumina (New England BioLabs, Ipswich, MA, USA) was used to create libraries from 250 ng of total RNA with adapters and PCR primers from New England BioLabs. The NEBNext Poly(A) Magnetic Isolation Module (New England BioLabs) was used for mRNA enrichment. The cDNA synthesis was performed using the NEBNext RNA First Strand Synthesis and NEBNext Ultra Directional RNA Second Strand Synthesis Modules (New England BioLabs). Library quantification was performed using the Kapa Illumina GA and Revised Primers-SYBR Fast Universal kit (Kapa Biosystems, Potters Bar, UK). Fragment size was determined using the LabChip GX (PerkinElmer, Waltham, MA, USA). Libraries were normalized, pooled, denatured in 0.05N NaOH, and then neutralized using HT1 buffer. The pooled libraries were loaded on NovaSeq S4 l (Illumina, San Diego, CA, USA) lanes at concentration of 225 pM, following the manufacturer’s Xp protocol. Sequencing was performed for 2 × 100 cycles (paired-end mode) with a phiX library as control. RTA v3.4.4 (Illumina) was used for base calling followed by bcl2fastq2 v2.20 (Illumina) to demultiplex samples and generate FASTQ reads.

### 2.3. Quality Control, Annotation to Reference Genomes, and Quantification of Counts

The RNA-seq StringTie workflow was followed from GenPipes v3.1.5 (https://bitbucket.org/mugqic/genpipes/src/master/pipelines/rnaseq/, accessed on 5 August 2020) [25]. Adaptor sequences and low-quality scoring bases from sequenced reads (Phred score < 30) were trimmed using Trimmomatic v0.36 [26]. The resulting reads from the chickens and ducks were aligned to *Gallus gallus* GRCg6a genome (release 98) and *Anas platyrhynchos platyrhynchos* CAU_duck1.0 genome (release 98), respectively, using STAR (Spliced Transcripts Alignment to a Reference) software, v2.5.3a [27]. For the Muscovy ducks, the *Anas platyrhyncos platyrhyncos* (Pekin duck) genome was used as this was annotated in much greater detail than the recently released *Cairina moschata* genome (CaiMos1.0), allowing more accurate downstream analyses. Both reference genomes were retrieved from Ensembl (https://www.ensembl.org, accessed on 21 August 2020 [28]). Read counts for both species were obtained using HTseq v0.6.1p1 [29].

### 2.4. Principal Component and Differential Gene-Expression Analysis

Principal component analysis (PCA) was carried out to evaluate the contribution of infection status (i.e., treatment) and week post-infection (i.e., time) to explaining the variability of the dataset. PCA plots were created using DESeq2 on the transformed gene-expression estimates using the ggplot2 R library. Venn diagrams were created using the R package, ggVenn v0.1.8.

Differential gene expression (DGE) analysis was conducted by comparing relative gene expression of infected birds with the time-matched control group using the DESeq2 R package, v1.30.0 [30]. First, we examined the total number of differentially expressed genes (tDEGs), as defined by adjusted *p*-values < 0.05 and absolute log_2_ fold change > 0 (|log_2_FC| > 0), which is equivalent to absolute fold change > 1 (|FC| > 1). Further, we evaluated the genes that were highly differentially expressed (hDEG), as defined by adjusted *p* values < 0.05 and absolute log_2_ fold change > 2 (|log_2_FC| > 2), which is equivalent to absolute fold change > 4 (|FC| > 4). The high threshold (|log_2_FC| > 2) was used to increase reproducibility, since replicability of gene calling is correlated with the magnitude of their differential expression [31,32]. The *p*-values were corrected using the Benjamini-and-Hochberg method for controlling the false-discovery rate [33].

### 2.5. Functional Analysis of DEG using Gene Ontology (GO) Terms and Kyoto Encyclopedia of Genes and Genomes (KEGG) Pathway Enrichment

GO terms and KEGG pathway enrichment analysis were used to investigate the functional significance of the hDEG (|log_2_FC| > 2). GO enrichment analysis was performed using the GOseq R package, v1.42.0 [34]. GO terms with Benjamini-and-Hochberg-adjusted *p*-values < 0.05 were considered significantly enriched. KEGG pathway enrichment analysis was performed using the g:Profiler web server version e105_eg52_p16_e84549f [35]. For chickens, the g:GOSt function from the g:Profiler web server was used to perform KEGG enrichment analysis with *Gallus gallus* selected as the organism and 0.05 selected as the g:SCS significance threshold. For ducks, KEGG pathway enrichment analysis was performed indirectly, due to limited pathway annotation for duck genes, as follows. First, duck hDEGs were converted to chicken ortholog identifiers using the g:Orth function on g:Profiler. Next, the g:GOSt function was used to perform KEGG pathway enrichment analysis on the chicken orthologs of duck genes, with *Gallus gallus* selected as the organism and 0.05 selected as the g:SCS significance threshold.

## 3. Results

### 3.1. Microscopic Pathology and Magnitude of Virus Replication in the Brains of Chickens and Ducks Infected with ABBV-1 upon Intracranial Administration

The brains of Muscovy ducks and White Leghorn chickens were evaluated for histopathology and quantification of RNA virus copies by RT-qPCR. The group means are reported in Table 1, and individual bird data are reported in Supplemental Table S1.

No birds developed neurological signs during the course of the experiment, with the exception of a single chicken that underwent emergency euthanasia and was not sampled for the RNAseq analysis [15,16]. When present, inflammatory lesions were similar in both species and were characterized by lymphocytic cuffs in the perivascular spaces, occasionally spilling into the adjacent neuroparenchyma. No evidence of neuronal necrosis was observed. The ducks showed a high intensity of inflammation at 4 wpi (average, 3), which decreased at 12 wpi (average, 2). The chickens showed no inflammation at 4 wpi, while at 12 wpi, they had marked inflammation (average, 3.75). The magnitude of the viral replication, as expressed by RNA copy number per 150 ng of total tissue RNA, was similar in the ducks at the two time points, while it was higher in the chickens at 12 wpi compared to at 4 wpi.

### 3.2. Overview of Sequencing, Trimming, and Alignment Analysis of Duck- and Chicken-Brain Tissues Infected with ABBV-1 or Mock Infected

The number of raw reads for the duck samples (n = 28) fell between 66,851,188 and 136,268,072 (median, 98,259,110). After the adaptor sequences and low-quality score-containing bases (Phred score < 30) were trimmed from the reads using Trimmomatic [26], more than 99.90% of the raw reads remained as surviving reads in each sample. When the surviving reads were aligned to the *Anas platyrhynchos platyrhynchos* reference genome (CAU_duck1.0.98), between 72.64% and 80.56% (median, 76.76%) of the reads were aligned, resulting in a mean coverage per base of between 38.44 and 80.03 (median, 53.61). The number of genes covered by the reads was between 9703 and 10,080 (median, 9877) (Supplemental Table S2).

The number of raw reads for the chicken samples (n = 28) fell between 82,253,152 and 170,553,534 (median, 101,211,921). After the adaptor sequences and low-quality score-containing bases (Phred score < 30) were trimmed from the reads using Trimmomatic [26], more than 99.90% of the raw reads remained as surviving reads in each sample. When the surviving reads were aligned on the *Gallus gallus* reference genome (Gallus gallus.GRCg6a.98), between 91.25% and 96.60% (median, 94.57%) of the reads were aligned (Supplemental Table S3), resulting in a mean coverage per base of between 49.75 and 101.57 (median, 67.06). The number of genes covered by the reads was between 11,697 and 11,990 (median, 11,820.50) (Supplemental Table S3).

### 3.3. Inflammation Is the Main Driver of Altered Transcriptional Profiles in ABBV-1-Infected Duck and Chicken Brains, as Shown by PCA and DGE Analyses

#### 3.3.1. PCA Analysis

**Figure 1:**
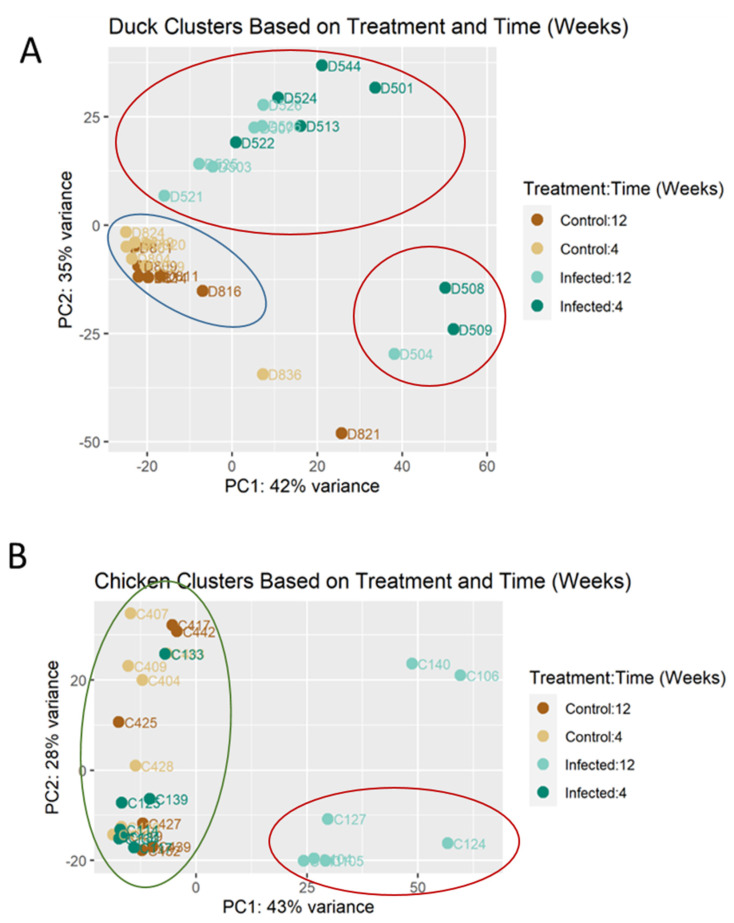
Principal component analysis (PCA) plot of Muscovy duck (**A**) and chicken (**B**) cohorts infected with ABBV−1. Light brown and dark brown represent control birds from 4 and 12 wpi, respectively. Light blue and dark bluerepresent infected birds from 4 and 12 wpi, respectively. The alphanumeric code adjacent to each data point represents a single bird identifier. For each species, there were 7 birds per treatment per time point. Red circle shows clustering of infected animals and blue circle shows clustering of control animals. Green circle shows mixture of infected and control animals.

In the duck cohort, as shown by the PCA plots (Figure 1A), there was a separation between most of the control and infected ducks, with PC1 and PC2 accounting for 42% and 35% of the variance, respectively. The control birds displayed a tight grouping, while the infected birds had a more dispersed variance. While the timing of the infection could not clearly separate the birds sampled at 4 and 12 wpi, the ducks at 4 wpi grouped farthest away from the control birds along the PC1 axis. In the chicken cohort, PC1 and PC2 accounted for 43% and 28% of the total variance, respectively (Figure 1B). There was a clear separation between the control group and the infected birds at 12 wpi along the PC1 axis; however, the infected birds at 4 wpi clustered with the controls. Considering the pathology data, the PCA analysis suggested that differences in the transcriptomic profiles between the infected and control birds paralleled the presence of inflammation, as only groups with mononuclear inflammation clustered separately from the controls. On the other hand, these data suggest that ABBV-1 infection alone, without inflammation, did not sufficiently alter the transcriptomic profiles compared to the uninfected controls, as seen in the chickens at 4 wpi.

#### 3.3.2. DGE Analysis

**Figure 2:**
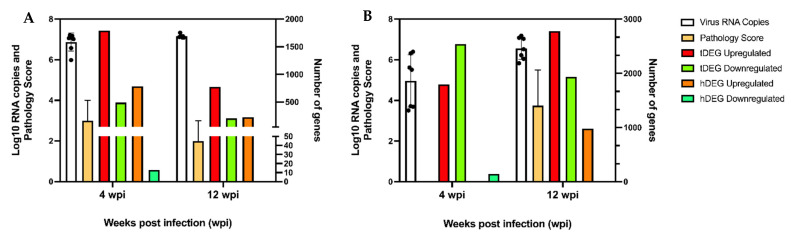
Overview of viral RNA copies and pathology scores in the brains of ducks and chickens alongside total and highly differentially expressed genes in those tissues. In (**A**), data are presented for Muscovy ducks at 4 wpi and 12 wpi. In (**B**), data are presented for chickens at 4 wpi and 12 wpi. The left *y*-axis shows both the log_10_ RNA copies and pathology scores. The right *y*-axis shows the number of differentially expressed genes. The *x*-axis shows the weeks post-infection. tDEG stands for total differentially expressed genes and hDEG stands for highly differentially expressed genes. The viral-RNA and pathology-score data used in this figure are adapted from [15,16].

A differential gene-expression analysis was performed separately for each species, to compare transcript-expression levels between infected and age-matched control birds. A summary of the number of differentially expressed genes for both species is reported in Table 2; furthermore, contextualized data with the results from the experimental challenge are represented in Figure 2. The ducks had a greater number of tDEGs at 4 wpi (2283) compared to 12 wpi (979) (Table 2), with 78.4% and 79.0% of the tDEGs being upregulated at 4 wpi and 12 wpi, respectively. Of the 2283 tDEGs at 4 wpi, 1313 were known, and 970 were novel genes. Of the 979 tDEGs at 12 wpi, 479 were known genes and 500 were novel genes.

**Figure 3:**
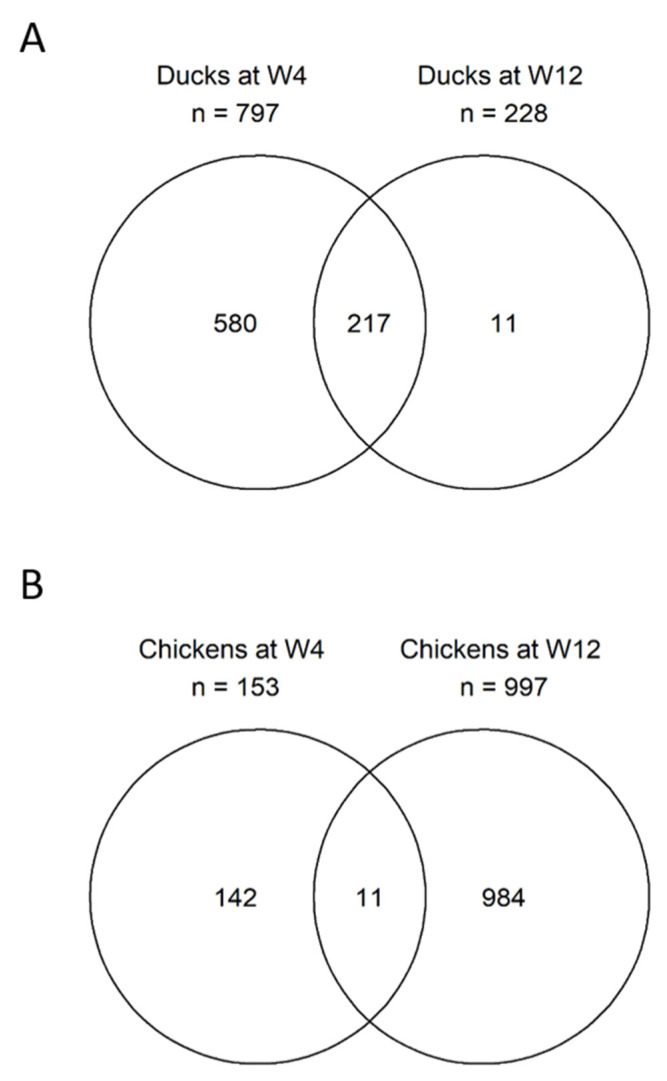
Venn diagram showing proportions of highly differentially expressed genes (hDEGs) between 4 and 12 weeks post-infection (wpi), in both Muscovy ducks and chickens. In (**A**), hDEGs in the brains of ABBV-1-infected Muscovy ducks at 4 wpi were compared with those at 12 wpi. The majority of hDEGs occurred at 4 wpi, and 217 hDEGs were shared between 4 and 12 wpi. In (**B**), the hDEGs in the brains of ABBV-1-infected chickens at 4 wpi were compared with those at 12 wpi. The majority of hDEGs occurred at 12 wpi, and only 11 hDEGs were shared between the two time points.

The number of hDEGs was 797 at 4 wpi (34.9% of tDEGs) and 228 at 12 wpi (23.5% of tDEG). Of these, 217 hDEGs were shared between 4 and 12 wpi (Figure 3A). Nearly all (98.4%) and 100% of the hDEGs were upregulated at 4 and 12 wpi (Table 2), respectively, and all of the 217 DEGs shared between the time points were also upregulated.

The chickens had 4328 tDEGs at 4 wpi and 4718 tDEGs at 12 wpi. At 4 wpi, more genes were downregulated (58.5%), while at 12 wpi, this pattern reversed, and there were more upregulated genes (58.9%) (Table 2). Of the 4328 tDEGs at 4 wpi, 3325 were known genes and 1003 were novel genes. Of the 4718 tDEGs at 12 wpi, 3506 were known genes and 1213 were novel genes. The number of hDEGs was drastically reduced, constituting only 3.5% (n, 153) and 21.1% (n, 997) of the tDEGs at 4 ad 12 wpi, respectively. At 4 wpi, most (94.8%) of the hDEGs were downregulated, while at 12 wpi, 98.2% of the hDEGs were upregulated. Interestingly, only 58.9% of the tDEGs were upregulated at this time point (Table 2). Only 11 of the hDEGs were shared between 4 and 12 wpi (Figure 3B), including both up- and downregulated genes.

In summary, there were differences in the global differential gene-expression patterns of the duck and chicken brains in response to ABBV-1 infection, mostly in association with the degree of encephalitis. For the ducks, the number of tDEGs and hDEGs was highest at 4 wpi, when inflammation was high, and decreased at 12 wpi, when lower inflammation-severity scores were registered. Regardless of the time point, most of the hDEGs were upregulated in the ducks. For the chickens, this difference was noted mainly for the hDEGs, which dramatically increased from 4 wpi, when no inflammation was detected, to 12 wpi, the time of high inflammation. Notably, the majority of the tDEGs (58.5%) and hDEGs (94.8%) were downregulated in the chickens at 4 wpi, when infection was established, but inflammation was not detected.

### 3.4. Highest-Ranking hDEGs (|log_2_FC| > 2) in Duck and Chicken Brains in Response to ABBV-1 Infection Are Associated with Immune Functions, Regulation of Gene Expression, and the Cell-Membrane Location

We further examined the 20 highest-ranking known and unknown (novel) hDEGs in the duck and chicken brains at 4 and 12 wpi. The known genes, along with their descriptions (as provided in the Ensembl database), are reported in Table 3 and Table 4; the unknown (novel) genes and either their descriptions (as provided on the Ensembl database) or their corresponding protein names (as provided in the UniProt database) are listed in Supplemental Tables S4 and S5. Not every group produced at least 20 hDEGs. The genes of interest are mentioned below.

#### 3.4.1. Ducks

At 4 wpi, there were more than twenty upregulated hDEGs, but only five downregulated hDEGs (Table 3). Fourteen of the 20 highest-ranking upregulated hDEGs are associated with immune functions: *LAG3*, *CXCR5*, *JCHAIN*, *CTLA4*, *AICDA*, *TRAT1*, *SERPING1*, *IFNG*, *XCR1*, *ZAP70*, *IL22RA2*, *VPREB3*, *PAX5*, and *CD79B*. Four are associated with the regulation of gene expression: *SPIC*, *HOXA4*, *PLAC8*, and *APOBEC1* (Table 3). Of the five downregulated hDEGs, three are associated with the cell-membrane region (*CDH15*, *TMEM233*, and *ATP6V0D2*), and two are associated with the regulation of gene expression (*ZIC1* and *ZIC4*) (Table 3). Considering the novel genes, there were more than 20 upregulated hDEGs and only 8 downregulated hDEGs (Supplemental Table S4). Sixteen of the twenty highest-ranking upregulated novel genes are associated with immune function, as evidenced by the generic protein names, such as ‘Ig-like domain-containing protein’ or ‘SCY domain-containing protein’, on the UniProt database (Supplemental Table S4). Eight of the downregulated genes are labeled as ‘long non-coding RNA’ in the Ensembl database (Supplemental Table S4).

At 12 wpi, there were more than 20 upregulated hDEGs, but no downregulated hDEGs (Table 3). Sixteen of the twenty highest-ranked upregulated hDEGs are associated with immune functions: *CTLA4*, *JCHAIN*, *OASL*, *SERPING1*, *LAG3*, *EPSTI1*, *RSAD2*, *CALHM6*, *CD6*, *DHX58*, *MX1*, *ZAP70*, *IRF7*, *DDX60*, *DTX3L*, and *B2M*. Three are associated with the regulation of gene expression: *PLAC8*, *7SK*, and *PARP10*. Considering the novel genes, there were more than 20 upregulated hDEGs, but no downregulated hDEGs (Supplemental Table S4). Fourteen out of these twenty genes are associated with immune function, with generic protein names such as ‘Ig-like domain-containing protein’ or ‘SCY domain-containing protein’, on the UniProt database (Supplemental Table S4).

#### 3.4.2. Chickens

At 4 wpi, there were only five upregulated hDEGs but more than twenty downregulated hDEGs (Table 4). Of the upregulated genes, four are associated with immune functions: *IRF4*, *IFIT5*, *RSAD2*, and *LYG2*. For the downregulated genes, two are associated with responses to pathogens (*VWCE* and *NOS1*) and eight are associated with the cell-membrane location: *SLC16A8*, *BAIAP2L2*, *CGN*, *KCNH6*, *SLC10A4*, *GPRC5C*, *KCNK3*, and *DMP1*. Considering the novel genes, there were only three upregulated hDEGs, but more than twenty downregulated hDEGs (Supplemental Table S5). For the three upregulated genes, either they were labeled as ‘long non-coding RNA’ on the Ensembl database, or their proteins were labeled as ‘uncharacterized protein’ on the UniProt database. For the twenty highest-ranked downregulated hDEGs, three are associated with immune functions: *ENSGALG00000053055* (B30.2/SPRY domain-containing protein), *ENSGALG00000054795* (TED_complement domain-containing protein), and *ENSGALG00000007740* (Wiskott–Aldrich-syndrome-protein-family member). Five are labeled as ‘long non-coding RNA’, according to the Ensembl database, and four encode ‘uncharacterized proteins’, according to the UniProt database. The rest of the novel genes could not be characterized further.

At 12 wpi, there were more than 20 upregulated hDEGs, but only 10 downregulated hDEGs (Table 4). Fifteen of the twenty highest-ranking upregulated hDEGs are associated with immune functions: *IFNG*, *IL18RAP*, *JCHAIN*, *LAG3*, *CTLA4*, *CCL5*, *CXCL13L3*, *CRTAM*, *CCL1*, *XCL1*, *CCL19*, *POU2AF1*, *CXCL13L2*, *GZMA*, and *AICDA* (Table 4). Of the ten downregulated genes, three are associated with the cell-membrane region: *BAIAP2L2*, *KCNH8*, and *KCNK3*. Considering the novel genes, there were more than 20 upregulated hDEGs, but only 8 downregulated hDEGs (Supplemental Table S5). Nineteen of the twenty highest-ranked upregulated genes are associated with immune functions and possess generic protein names, such as ‘Ig-like domain-containing protein’ or ‘Immunoglobulin lambda like polypeptide 1′, on the UniProt database (Supplemental Table S5). Of the downregulated genes, one (*ENSGALG00000029168*) encodes a ‘collectrin domain-containing protein’, and four are labeled as ‘long non-coding RNA’ on the Ensembl database. One novel gene (*ENSGALG00000043906*), which was downregulated at both 4 wpi and 12 wpi, encodes the protein ‘chicken D-serine dehydratase’. The rest of the novel genes could not be characterized further.

### 3.5. ABBV-1 Differentially Regulates Long Non-Coding RNA (lncRNA) Expression in Both Duck and Chicken Brains

The examination of the novel hDEGs by searching each against the Ensembl database (Ensembl release 106: April 2022) showed that many were lncRNAs. A similar examination of the known hDEGs showed no lncRNAs among them. The total number of highly differentially expressed novel lncRNAs is reported in Table 5, and the 20 highest-ranked are shown in Supplemental Table S6. In the ducks at 4 wpi, 95 lncRNAs were highly differentially expressed, of which 87 were upregulated and 8 were downregulated. At 12 wpi, 22 lncRNAs were highly differentially expressed, all of which were upregulated. In the chickens at 4 wpi, only 13 lncRNAs were highly differentially expressed, of which 2 were upregulated and 11 were downregulated. At 12 wpi, 123 lncRNAs were highly differentially expressed, of which 119 were upregulated and 4 were downregulated (Table 5). Overall, the differential expression of the lncRNAs followed the transcriptional dynamics of the other genes and the magnitude of encephalitis, with the highest upregulation in the ducks at 4 wpi and in the chickens at 12 wpi. Some of the lncRNAs showed upregulation of up to 7.33- and 7.95-log_2_ fold in the ducks and chickens, respectively. The functional gene ontology (GO) and Kyoto Encyclopedia of Genes and Genomes (KEGG) pathway analyses of these novel lncRNAs using g:Profiler (version e106_eg53_p16_65fcd97) showed no enrichment of either the GO terms or the KEGG pathways.

### 3.6. Gene Ontology (GO) Analysis of hDEGs Demonstrates Enrichment of GO Terms Broadly Associated with Immune Functions

#### 3.6.1. Ducks

**Figure 4:**
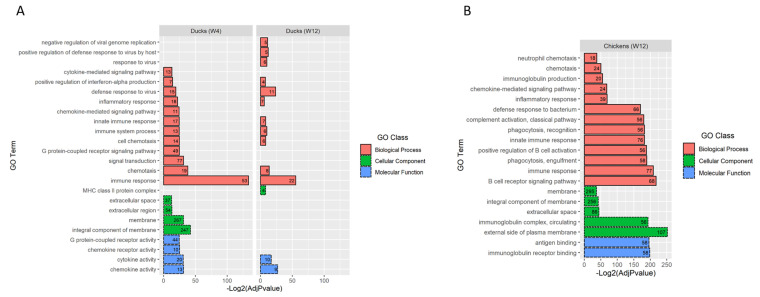
Gene ontology (GO) analysis of Muscovy duck and chicken brains infected with ABBV−1 at 4 and 12 weeks post-infection (wpi). In (**A**), the 20 highest-ranked (out of 59) and 14 (out of 14) enriched GO terms are shown for the Muscovy ducks sampled at 4 wpi (left graph) and 12 wpi (right graph), respectively. In (**B**), the 20 highest-ranked (out of 107) significantly enriched GO terms are shown for the chickens sampled at 12 wpi. Chicken brains sampled at 4 wpi did not produce any significantly enriched GO terms. The significance of the GO terms’ enrichment is represented as −log_2_ of the adjusted *p*-values on the *x*-axis. The GO categories are labeled and grouped by colors (*y*-axis): red bars represent biological processes, green bars represent cellular components, and blue bars represent molecular function. The numbers inside the bars represent the numbers of differentially expressed genes per GO term.

The enrichment analysis of the hDEGs in the duck brains at 4 wpi showed that there was a total of 59 significantly enriched GO terms: 38 in ‘biological process’, 9 in ‘cellular component’, and 12 in ‘molecular function’. The 20 highest-ranked enriched GO terms are shown in Figure 4A. Twelve of these are broadly associated with immune functions, anti-pathogen defense, and transmission signaling (‘biological process’ class); the terms with the highest number of hits included ‘signal transduction’ (n, 77), ’immune response’ (n, 53), and ‘G protein-coupled receptor signaling pathway’ (n, 49). The ‘cellular component’ class included the two most enriched GO terms: ‘membrane’ (n, 267) and ‘integral component of membrane’ (n, 247). The ‘molecular function’ class included enriched GO terms associated with cell signaling (Figure 4A), likely as part of the activated immune response.

At 12 wpi, there were only 14 significantly enriched GO terms: 11 in ‘biological process’, 1 in ‘cellular component’, and 2 in ‘molecular function’ (Figure 4A). Overall, the terms were less enriched compared to at 4 wpi, and tended to have a lower statistical significance. These GO terms are broadly associated with immune functions, anti-pathogen defense, or cell signaling. The membrane- and extracellular-space-associated GO terms, markedly enriched at 4 wpi, were no longer so at 12 wpi.

#### 3.6.2. Chickens

The enrichment analysis of the hDEGs in the chicken brains at 4 wpi showed no significantly enriched GO terms, while at 12 wpi, there were a total of 107 significantly enriched GO terms: 82 in ‘biological process’, 8 in ‘cellular component’, and 17 in ‘molecular function’. The 20 highest-ranked enriched GO terms are shown in Figure 4B. Sixteen of these are broadly associated with immune functions or anti-pathogen defense; the most enriched terms are associated with the humoral immune response and B-cell activation, such as ‘B cell receptor signaling pathway’ (n, 68), ‘antigen binding’ (n, 58), ‘immunoglobulin receptor binding’ (n, 58), ‘positive regulation of B cell activation’ (n, 56), ‘immunoglobulin complex, circulating’ (n, 56), and ‘immunoglobulin production’ (n, 20). The enrichment of the terms associated with the humoral immune response showed a marked difference from the ducks at both 4 and 12 wpi. Similarly to what was observed with the ducks at 4 wpi, four of the most enriched terms were classified in the ‘cellular component’ class and are broadly associated with the cell membrane or extracellular locations, including ‘membrane’ (n, 295), ‘integral component of membrane’ (n, 256), ‘external side of plasma membrane’ (n, 107), and ‘extracellular space’ (n, 86).

### 3.7. Kyoto Encyclopedia of Genes and Genomes (KEGG) Pathway Analysis of hDEG Demonstrates Enrichment of Pathways Associated with Immune Functions or Viral Infections

The KEGG pathway analysis of the hDEGs for the chicken brains at 4 wpi did not show any enrichment. The KEGG pathway analysis of the hDEGs for the ducks at 4 wpi, the ducks at 12 wpi, and the chickens at 12 wpi showed the enrichment of seven common pathways (Table 6) between all three groups. These were ‘cytokine-cytokine receptor interaction’, ‘toll-like receptor signaling pathway’, ‘herpes simplex virus 1 infection’, ‘cell adhesion molecules’, ‘influenza A’, ‘NOD-like receptor signaling pathway’, and ‘phagosome’. Among these pathways, the highest significance was observed in the ducks at 4 wpi and the chickens at 12 wpi, probably as a reflection of the inflammatory reactions in the brains. Five additional pathways were not common to all the groups and were only enriched in some (Table 6). Overall, except for the ‘cell adhesion molecules’ and ‘neuroactive ligand-receptor interaction’ pathways, all of the significantly enriched KEGG pathways are directly associated with immune functions or responses to virus infections.

## 4. Discussion

We evaluated the transcriptomic profiles of aquatic bird bornavirus -1 (ABBV-1) in the infected brains of the Muscovy ducks and chickens at 4 and 12 wpi. We opted for these two time points as 4 wpi was the earliest time point at which all the birds were infected and had a relatively high magnitude of viral RNA copies in their brain tissues. The last time point was included to model chronic infection, as seen in the field. In choosing 4 wpi as our first time point in the analysis, we decreased our ability to assess transcriptomic changes that were not superimposed with inflammation (see below). Nonetheless, the use of brains with established infection was considered the most stringent inclusion criterion for this study. Moreover, age-matched controls were included to account for transcriptomic changes associated with the fast growth of poultry during their first few weeks of age.

For the entire data set, the number of aligned (mapped) reads from the samples was between 53,167,653 (lowest) and 157,453,838 (highest). The number of aligned reads per sample in the data set was well above the recommended number, approximately 36 million, for the accurate determination of the abundance in 80% of the genes [36]. The percentage of aligned reads for the chickens was between 91% and 97%, and for the ducks, it was between 70% to 81%. The reduced alignment for the Muscovy-duck samples was probably due to the use of the *Anas platyrhynchos platyrhynchos* (Pekin duck) reference genome instead of the *Cairina moschata* reference genome. The latter was not used because it is relatively recent and poorly annotated compared to the Pekin-duck reference genome, thus limiting downstream analyses. Nonetheless, a percentage of aligned reads between 70% and 90% is acceptable for further analysis [37]. The use of 7 biological replicates (i.e., 7 brain samples) per experimental unit (group/time point) is in line with the recommend minimum of 6 replicates per condition to identify differentially expressed genes [38].

Changes in the transcriptomic profiles in response to ABBV-1 infection appeared earlier in the duck brains than in those of the chickens, as shown by the PCA and DGE analyses. In the PCA analysis, the infected duck-brain tissues were clearly separated from control-brain tissues both at 4 and 12 wpi, whereas the infected chicken-brain tissues were only separated from the controls at 12 wpi. While the chicken brains had a greater number of tDEGs than the ducks at 4 wpi (4328 vs. 2283), when considering hDEG, only 153 genes were upregulated in the chicken brains (3.5% of tDEG), compared to 797 in those of the ducks (34.9% of the tDEGs). Furthermore, by 12 wpi, the transcriptome response of the ducks decreased to 979 tDEGs (228 hDEGs), while in chickens, it increased to 4718 tDEGs (997 hDEGs). This pattern correlated with the development of inflammation in the brains. The ducks had a higher brain-pathology score at 4 wpi (3.0 score) than at 12 wpi (2.0 score), while in the chickens, inflammation did not develop at 4 wpi (0 score) but did at 12 wpi (3.75 score) [15,16]. The functional evaluation of the upregulated genes further indicated that the transcriptomic changes in the brains of the ducks at both time points and in those of the chickens at 12 wpi were driven by inflammation, as most (≥70%) of the 20 most upregulated hDEGs were associated with immune functions. The reduced ABBV-1 viral load in the chicken brains could explain the lower degree of inflammation and the less-significant transcriptomic changes. In fact, at 4 wpi, the duck brains had an average of 10^6.87^ viral RNA copies per 150 ng of total RNA, while the chicken brains had approximately 10^4.97^ [15,16]. By 12 wpi, the average viral RNA copy in the duck brains remained relatively steady at 10^7.15^, while it increased to 10^6.56^ in those of the chickens, where it was approximately 39 times higher than at 4 wpi. The lower ABBV-1 replication in the chickens relative to the ducks was not surprising, since the same was also true in vitro, where ABBV-1 was propagated less efficiently in chicken compared to duck cell lines [17,39]. Moreover, the lower concentration of inoculum in the chickens might have played a role.

The chickens at 4 wpi had a much greater proportion of downregulated tDEGs (58.5%) and hDEGs (94.8%) compared to the chickens at 12 wpi (41.1% of tDEGs and 1.8% of hDEGs) and the ducks at both 4 wpi (21.6% of tDEGs and 1.6% of hDEGs) and 12 wpi (21.0% of tDEGs and 0% of hDEGs). Numerous (8/20) downregulated hDEGs in the chicken brains at 4 wpi were associated with cell-membrane regulation, which suggests that ABBV-1 infection alone, without superimposed inflammation, may alter cell-membrane homeostasis. However, this differential gene expression was not sufficient to separate the infected chickens at 4 wpi from the controls in the PCA analysis and did not significantly enrich the GO terms or KEGG pathways. Furthermore, in the other groups, some of the downregulated hDEGs were associated with the cell membrane or cell-membrane/extracellular-matrix region, although this proportion varied (3/5 for the ducks at 4 wpi, 0 for the ducks at 12 wpi, and 3/10 for the chickens at 12 wpi). This may indicate a separate contribution of ABBV-1 infection to transcriptomic changes, distinct from inflammation. In a microarray experiment using brains from rats infected with BoDV-1 [18], cell-membrane/extracellular-matrix-region-associated genes, such as Ca-transporter ATPase3, Na/K transporting ATPase beta 1 subunit, Ras-guanine nucleotide release/exchange factor p140, and vacuolar ATP synthase 16-kDa proteolipid subunit (*ATP6V0C* (ATPase H+ transporting V0 subunit C)), were downregulated [18], supporting our observations. For example, the *ATP6V0D2* (ATPase H+ transporting V0 subunit d2) gene was highly downregulated in ducks at 4 wpi. The other downregulated genes in our study included *CHRNA2* (Cholinergic receptor nicotinic alpha 2 subunit), *KCNH6* (potassium voltage-gated channel subfamily H member 6), *SLC10A4* (solute carrier family 10 member 4), *CBLN1* (cerebellin 1 precursor), *NOS1* (nitric oxide synthase 1), and *SHISA6* (shisa family member 6), which are associated with neurotransmission and synaptic physiology, suggesting the downregulation of these neuronal activities by ABBV-1. Furthermore, the BoDV-1 infection of rat neurons was shown to impair neuronal remodeling and synaptic plasticity [22,40].

When inflammation was present (ducks at 4 and 12 wpi, and chickens at 12 wpi), there was a marked upregulation of immune-check-point genes in both species; specifically, *LAG3* (lymphocyte activating 3) and *CTLA4* (cytotoxic T-lymphocyte-associated protein 4). LAG3 is a membrane receptor protein expressed on T cells, which binds to the MHC class II receptor, preventing its binding with the T-cell receptor (TCR) and CD4 [41]. This leads to the suppression of TCR signaling and, consequently, the suppression of CD4^+^ and CD8^+^ T cell activation. CTLA4 is a membrane receptor protein that is constitutively expressed in regulatory T cells [42]. It can competitively bind to CD80/86 on antigen-presenting cells (APCs), preventing its interaction with the CD28 on T cells, leading to the inhibition of CD4^+^ T-cell activation [42]. This suggests that ABBV-1-infected brain tissues may develop a mechanism to control neuroinflammation and prevent the development of functional T cells, despite their inability to prevent the accumulation of lymphocytes in the neuroparenchyma. This could explain the lack of neurological signs despite the morphological evidence of encephalitis, as well as the lack of neuronal necrosis. By contrast, the neuroinflammation caused by other orthobornaviruses, such as BoDV-1, variegated squirrel bornavirus 1 (VSBV-1), and parrot bornavirus-2 (PaBV-2), can result in disease development driven by T-cell-dominated immunopathogenesis, often resulting in histological evidence of neuronal degeneration [2,14,43,44,45]. In one genome-wide RNAseq study, the BoDV-1 infection of newborn rats led to neurological signs in the animal, and the transcriptomic analysis of their brains showed the upregulation of inflammatory cytokines [23]. Additionally, KEGG pathways similar to those enriched in duck and chicken brains infected with ABBV-1, such as ‘cytokine-cytokine receptor interaction’, ‘toll-like receptor signaling pathway’, ‘NOD-like receptor signaling pathway’, and ‘intestinal immune network for IgA production’, were enriched in the rat brains [23]. However, very few studies have evaluated the transcriptomic profiles of viral infection in avian brains. In ducks infected with highly pathogenic H5N1, the development of neurological signs was correlated with the upregulation of immune-associated genes, such as PRRs, IFNs, and cytokines, with no upregulation of regulatory genes, such as *LAG3* or *CTLA4* [46]. Future studies should evaluate the expression of immune-checkpoint genes or the relative amounts of regulatory T cells in the perivascular lymphocytic cuffs of the nervous tissue, as possible drivers of asymptomatic disease in orthobornavirus infection.

Numerous novel lncRNAs were highly differentially expressed in the brains of the ducks and chickens infected with ABBV-1; however, their functions are unknown, as these genes did not yield any significantly enriched GO terms or KEGG pathways. This is likely to have been a consequence of the only partial annotation of lncRNAs in the respective reference genomes of ducks (CAU_duck1.0) and chickens (GRCg6a). Although the function of lncRNAs in cells is broad, with roles in many diverse biological processes [47,48], most of the lncRNAs in our experimental cohort appeared to correlate with the severity of inflammation. In fact, the differential regulation of the lncRNAs was higher in the ducks at 4 wpi and in the chickens at 12 wpi, and lower in the ducks at 12 wpi and in the chickens at 4 wpi. This suggests that the differential expression of the lncRNAs occurred in the inflammatory cells rather than the neuroparenchyma, which is consistent with the established role of lncRNAs in regulating the inflammatory response in mammals [49,50,51]. Nonetheless, some of the lncRNAs were differentially expressed either in the absence of inflammation (chicken 4 wpi), or when only mild inflammation was present (ducks 12 wpi), suggesting differential expression in virus-infected neurons or glial cells. A genome-wide microarray study of primary cultures of mouse cortical neurons showed the differential regulation of 3528 and 2661 lncRNAs upon infection with BoDV-1 Strain V and Hu-H1, respectively [24]. A functional analysis showed that these lncRNAs were involved in numerous processes, including metabolic and biological regulation, cell adhesion, endocytosis, cancer, and viral infections [24]. The relationship between lncRNAs and bornavirus infection is an interesting area of future investigation.

The GO analysis of both the ducks and the chickens produced many enriched GO terms, of which most (≥60%) of the 20 most enriched were broadly associated with immune functions or anti-pathogen defense, while the remainder were associated with the membrane or extracellular location. The enrichment of these GO terms seemed to correlate with the severity of the inflammation and dovetailed with the number of DEGs. The ducks at 4 wpi had more enriched GO terms (59) than the ducks at 12 wpi (14), and in the chickens, the GO terms were only enriched at 12 wpi, as no inflammation was observed at 4 wpi. The GO terms in the cellular-component class were broadly associated with the cell membrane and extracellular space and included some of the most enriched terms in the whole analysis (i.e., ‘membrane’, ‘integral component of membrane’, and ‘external side of the plasma membrane’). This may have been due, at least in part, to the links between some of the immune-associated genes and multiple GO terms. For example, the upregulated *CTLA4* in the duck brains at 4 wpi is linked to five GO terms that are both related to immune response and the plasma membrane (i.e., ‘immune response’, ‘integral component of membrane’, ‘membrane’, ‘B cell receptor signaling pathway’, and ‘external side of plasma membrane’), and the *IFNG* gene is linked to 10 GO terms. Several membrane-associated genes were downregulated in the brains of the chickens at 4 wpi; however, these did not produce significantly enriched GO terms, and it is unclear whether ABBV-1 alone caused alterations in the membrane homeostasis. When the inflammation in the duck brains at 12 wpi decreased, the enrichment of the terms associated with the membrane and extracellular space was drastically reduced, despite the fact that the birds did not have high levels of viral RNA.

One difference between the GO profiles of the ducks and the chickens was the enrichment of the terms associated with adaptive immunity. GO terms such as ‘immunoglobulin production’, ‘positive regulation of B cell activation’, ‘B cell receptor signaling pathway’, ‘immunoglobulin complex, circulating’, ‘antigen binding’, and ‘immunoglobulin receptor binding’ were enriched in the chickens, whereas the ducks had ‘MHC class II protein complex’ as the only GO term clearly associated with adaptive immunity. The severity of the inflammation in the chickens at 12 wpi was similar to or greater than that observed in the ducks at 4 wpi, and in both species, the amount of Pax-5-positive cells (B lymphocytes) in the perivascular cuffs was low (<~10–15%) [15,16]. Therefore, the reason why the chickens had a greater proportion of GO terms associated with B-cell activation is unknown, but it may be related to species-specific differences in the immune response to viral infection. The presence of GO terms associated with adaptive immunity agrees with the evidence of seroconversion against ABBV-1 N protein in both species [15,16].

The KEGG analysis showed the enrichment of 12 pathways in ducks at 4 wpi, 9 in ducks at 12 wpi, and 10 in chickens at 12 wpi. Seven common KEGG pathways were enriched between all the groups. As with the GO analysis, the KEGG pathway analysis showed the enrichment of pathways mainly related to immune functions or viral infections, with the highest significance in the groups with the most inflammation (the ducks at 4 wpi and the chickens at 12 wpi). Multiple pattern-recognition receptor (PRR) pathways were enriched, including the ‘toll-like receptor-signaling pathway’, ‘NOD-like receptor-signaling pathway’, ‘cytosolic DNA-sensing pathway’, and ‘RIG-I-like receptor-signaling pathway’. The ‘toll-like receptor-signaling pathway’, ‘NOD-like receptor-signaling pathway’, and ‘cytosolic DNA-sensing pathway’ were also enriched in the brains of the rats after infection with BoDV-1 [23], corroborating our findings. Some of the DEGs enriched by ABBV-1 infection in these pathways included numerous PRRs genes, such as *TLR1A* (binds di- and triacylated lipopeptides), *TLR3* (binds dsRNA), *TLR4* (binds bacterial lipopolysaccharide), *TLR7* (binds ssRNA), *DHX58* (also known as (aka) *LGP2* and binds to dsRNA), IFIH1 (encodes *MDA5* and regulated by *DHX58*), and *TMEM173* (aka *STING1* and binds DNA) [52,53,54,55,56,57]. While *TLR4* is commonly known to be activated by bacterial lipopolysaccharide [54], it can be activated by multiple RNA viruses in different families [58] and by BoDV-1 [23]. TMEM173 is a cytosolic DNA sensor that binds DNA but can also be activated by RNA viruses through multiple proposed mechanisms [57]. Therefore, our demonstration of *TLR4* and *TMEM173* upregulation in the context of ABBV-1 is not without precedent.

The activation of PRRs leads to signaling cascades of multiple pathways, involving several proteins. Facilitator genes, such *DDX60*, *TRIM25*, and *TRIM14* were differentially expressed in response to ABBV-1 infection and have important roles in either the activation or propagation of the signaling cascades [59,60,61,62]. Signal transductions eventually result in, but are not limited to, the activation of *IRF-7*, which then induces the expression of IFN-α/β [63]. IFN-α/β initiates the JAK-STAT signaling-cascade pathway by binding to IFN-α/β receptors (IFNAR) on the cell membrane [64]. In our study, ABBV-1 infection resulted in the differential expression of *IRF-7*, *IFN-A*, *STAT1*, and *STAT2*. Similarly, the BoDV-1 infection of rat brains led to the upregulation of *IRF-7* and *STAT1* transcripts [20] and KEGG enrichment of the JAK-STAT signaling pathway [23]. The IFN JAK-STAT pathway induces the expression of a group of proteins collectively called interferon stimulated genes (ISG), some of which possess antiviral activities [65]. The ISGs induced by ABBV-1 in both Muscovy ducks and chickens included *OASL*, *RSAD2* (aka viperin), and *MX1*, all of which possess antiviral activities [65]. Similarly, *Mx1* transcripts, *OASL2* transcripts, and Mx1 proteins were also previously found to be upregulated in mice embryos and rat brains infected with BoDV-1 [20,66].

The ‘intestinal immune network for IgA production’ KEGG pathway was enriched in both chickens at 12 wpi (*p*-value adj. 6.503 × 10^−22^) and ducks at 4 wpi (*p*-value adj. 4.814 × 10^−8^), corresponding with the time points of severe inflammation. However, the statistical significance level of this enrichment was much greater in the chickens at 12 wpi than in the ducks at 4 wpi, which correlates with the enriched GO terms in the chickens associated with B-cell development and humoral immunity. Therefore, IgA production against ABBV-1 proteins was probably stimulated in the chickens and ducks. The BoDV-1 infection of rats also led to the enrichment of the ‘intestinal immune network for IgA production’ KEGG pathway, along with the ‘antigen processing and presentation’ KEGG pathway [23]. This further demonstrated the capacity of bornavirus to induce IgA in hosts.

The ‘necroptosis’ (or inflammatory cell death) KEGG pathway was enriched in the brains of infected ducks at 4 wpi, when the inflammation was severe. The enrichment of this pathway was further supported by the enrichment of both the ‘influenza A’ and ‘herpes simplex virus 1 infection’ KEGG pathways, as these were two viruses known to modulate necroptosis [67]. Necroptosis possibly occurred in the lymphocytes, as suggested by the histological evidence of the perivascular lymphocytic cuffs, and the lack of histological evidence of damage in neurons or glial cells. Necroptosis could have limited the excessive accumulation of lymphocytes and may explain the lower degree of inflammation in the brains of ducks at 12 wpi, compared to at 4 wpi. Since the immunopathogenesis of bornavirus infection is driven by primed cytotoxic CD8+ T lymphocytes against infected cells [44,68,69,70,71], the necroptosis of cytotoxic lymphocytes could explain the lack of clinical signs seen in our experimental trial. This explanation, however, remains speculative. To corroborate this mechanism, the expression of necroptosis markers, such as mixed lineage kinase domain-like (MLKL) and receptor interacting protein kinase 1 (RIPK 1) and 3 (RIPK 3), in lymphocytes should be evaluated by immunohistochemistry or in situ hybridization in sections of ABBV-1-infected brains [72].

## 5. Conclusions

This study shows that the transcriptomic changes in the brains of both Muscovy ducks and chickens infected with ABBV-1 were driven by the development of non-heterophilic inflammation, as evidenced by histopathology. The majority of highly differentially expressed genes were associated with immune response and cytokine signaling. Additionally, numerous highly differentially expressed novel genes were determined to be lncRNAs. In the groups with high magnitudes of inflammation (the ducks at 4 wpi and the chickens at 12 wpi), the chickens had greater upregulation of the pathways associated with adaptive immune response and B-cell maturation than the ducks.

When infection was established, but inflammation was not developed, as in the case of the infected chickens at 4 wpi, the transcriptomic changes were dominated by downregulated genes; however, no functional pathways were significantly enriched. This suggests that the transcriptomic changes caused by ABBV-1 infection alone are of low magnitude, supporting the notion that bornaviruses are not cytolytic and can establish chronic infection without severe cellular derangement.

Separating infection from inflammation may be of no use in the context of animal studies. While in vivo studies are needed to model infection in the host, in vitro or ex vivo studies may be better suited to assessing changes in the global transcriptomic profiles caused by ABBV-1, without the confounding effect of superimposed inflammation.

## Figures and Tables

**Table 1:** Virus RNA copies and histological scores of the brains of Muscovy ducks and chickens infected with ABBV-1 by the intracranial route, assessed at 4 and 12 weeks post-infection (wpi).

	Muscovy Ducks ^1^		White Leghorn Chickens ^2^	
	4 wpi	12 wpi	4 wpi	12 wpi
ABBV-1 RNA copy number ^3^	6.87 ± 0.45	7.15 ± 0.08	4.97 ± 1.28	6.56 ± 0.55
Histology score ^4^	3 ± 0.6	2 ± 0.6	0	3.75 ± 1.07

^1^ Data adapted from Iverson et al. [15]. ^2^ Data adapted from Iverson et al. [16]. ^3^ log_10_ viral RNA copies/150 ng of tissue RNA, average ± standard deviation. ^4^ Median ± standard deviation of the semi-quantitative severity of microscopic inflammation (0–6).

**Table 2:** Total (tDEG) and highly (hDEG) differentially expressed genes in the brains of ABBV-1-infected Muscovy ducks and chickens.

Species, Time Point	tDEG * (Absolute log_2_FC ** > 0)			hDEG (Absolute log_2_FC > 2)		
	tDEG	Upregulated (%) ^	Downregulated (%)	hDEG #	Upregulated (%) ^	Downregulated (%)
Ducks, 4 wpi	2283	1791 (78.4%)	492 (21.6%)	797 (34.9%)	784 (98.4%)	13 (1.6%)
Ducks, 12 wpi	979	773 (79.0%)	206 (21.0%)	228 (23.5%)	228 (100%)	0 (0%)
Chickens, 4 wpi	4328	1794 (41.5%)	2534 (58.5%)	153 (3.5%)	8 (5.2%)	145 (94.8%)
Chickens, 12 wpi	4718	2781 (58.9%)	1937 (41.1%)	997 (21.1%)	979 (98.2%)	18 (1.8%)

* DEG = differentially expressed genes; ** log_2_FC = log_2_ fold change; ^ indicates the total number of tDEGs or hDEGs and the percentage of tDEGs or hDEGs that are upregulated or downregulated are in parenthesis; # indicates the total number of hDEGs and the percentage of tDEGs that are hDEGs are in parenthesis.

**Table 3:** Top 20 highly up- and downregulated known DEGs in the brains of ABBV-1-infected Muscovy ducks.

Highly Upregulated DEGs at 4 wpi			Highly Upregulated DEGs at 12 wpi		
Gene	log_2_FC	Description (as Provided on Ensembl)	Gene	log_2_FC	Description (as Provided on Ensembl)
*LAG3*	11.62	Lymphocyteactivating 3	*CTLA4*	8.16	Cytotoxic T-lymphocyte associated protein 4
*SPIC*	11.14	Spi-C transcription factor	*JCHAIN*	7.41	Joining chain of multimeric IgA and IgM
*HOXA4*	10.59	Homeobox A4	*OASL*	6.64	2,-5,-oligoadenylate synthetase like
*CXCR5*	10.28	C-X-C motif chemokine receptor 5	*SERPING1*	6.33	Serpin family G member 1
*JCHAIN*	10.25	Joining chain of multimeric IgA and IgM	*LAG3*	5.70	Lymphocyte-activating 3
*CTLA4*	9.40	Cytotoxic T-lymphocyte associated protein 4	*EPSTI1*	5.53	Epithelial stromal interaction 1
*PLAC8*	9.05	Placenta-associated 8	*RSAD2*	5.25	Radical S-adenosyl methionine domain containing 2
*AICDA*	9.01	Activation induced cytidine deaminase	*CALHM6*	5.00	Calcium-homeostasis-modulator family member 6
*TRAT1*	8.90	T-cell receptor-associated transmembrane adaptor 1	*PLAC8*	4.90	Placenta-associated 8
*SERPING1*	8.80	Serpin family G member 1	*CD6*	4.67	CD6 molecule
*IFNG*	7.97	Interferon gamma	*DHX58*	4.31	DExH-BOX HELICASE 58
*XCR1*	7.75	X-C motif chemokine receptor 1	*MX1*	4.31	MX dynamin like GTPase 1
*ZAP70*	7.54	Zeta chain of T-cell receptor associated protein kinase 70	*ZAP70*	4.18	Zeta chain of T-cell receptor-associated protein kinase 70
*IL22RA2*	7.51	Interleukin 22 receptor subunit alpha 2	*IRF7*	3.91	Interferon regulatory factor 7
*VPREB3*	7.48	V-set pre-B-cell surrogate light chain 3	*DDX60*	3.69	DExD/H-box helicase 60
*PAX5*	7.15	Paired box 5	*EFCAB3*	3.64	EF-hand calcium binding domain 3
*NMU*	7.03	Neuromedin U	*7SK*	3.57	7SK RNA
*APOBEC1*	6.76	Apolipoprotein B mRNA editing Enzyme catalytic subunit 1	*DTX3L*	3.53	Deltex E3 ubiquitin ligase 3L
*CD79B*	6.74	CD79b molecule	*B2M*	3.37	Beta-2-microglobulin
*GPR65*	6.61	G-protein-coupled receptor 65	*PARP10*	3.31	Poly(ADP-ribose) polymerase family member 10
**Highly Downregulated DEGs at 4 wpi**					
**Gene**	**log_2_FC**	**Description** **(as Provided on Ensembl)**			
*CDH15*	−5.87	Cadherin 15			
*TMEM233*	−3.19	Transmembrane protein 233			
*ATP6V0D2*	−2.42	ATPase H+ transporting V0 subunit d2			
*ZIC1*	−2.38	Zic family member 1			
*ZIC4*	−2.14	Zic family member 4			

**Table 4:** Top 20 highly up- and downregulated known DEGs in the brains of ABBV-1 infected chickens.

Highly Upregulated DEG at 4 wpi			Highly Upregulated DEG at 12 wpi		
Gene	log_2_FC	Description (as Provided on Ensembl)	Gene	log_2_FC	Description (as Provided on Ensembl)
*IRF4*	3.12	Interferon regulatory factor 4	*IFNG*	11.01	Interferon gamma
*GUCA1C*	2.50	Guanylate cyclase activator 1C	*IL18RAP*	10.23	Interleukin 18 receptor accessory protein
*IFIT5*	2.43	Interferon induced protein with tetratricopeptide repeats 5	*JCHAIN*	9.55	Joining chain of multimeric IgA and IgM
*RSAD2*	2.16	Radical S-adenosyl methionine domain containing 2	*LAG3*	9.36	Lymphocyte activating 3
*LYG2*	2.09	Lysozyme g2	*AVD*	9.23	Avidin
**Highly Downregulated DEG at 4 wpi**			*HAAO*	9.16	3-hydroxyanthranilate 3,4-dioxygenase
**Gene**	**log_2_FC**	**Description** **(as Provided on Ensembl)**	*GPR55*	9.01	G protein-coupled receptor 55
*VWCE*	−6.83	Von Willebrand factor C and EGF domains	*CTLA4*	8.37	Cytotoxic T-lymphocyte associated protein 4
*SLC16A8*	−6.00	Solute carrier family 16 member 8	*CCL5*	8.29	C-C motif chemokine ligand 5
*BAIAP2L2*	−5.39	BAR/IMD domain containing adaptor protein 2 like 2	*CXCL13L3*	8.24	C-X-C motif chemokine ligand 13-like 3
*CGN*	−5.10	Cingulin	*CRTAM*	8.20	Cytotoxic and regulatory T cell molecule
*CHRNA2*	−4.96	Cholinergic receptor nicotinic alpha 2 subunit	*CCL1*	7.94	C-C motif chemokine ligand 1
*CBLN1*	−4.86	Cerebellin 1 precursor	*XCL1*	7.62	X-C motif chemokine ligand 1
*KCNH6*	−4.75	Potassium voltage-gated channel subfamily H member 6	*CCL19*	7.43	C-C motif chemokine ligand 19
*LZTS3*	−4.33	Leucine zipper tumor suppressor family member 3	*POU2AF1*	7.39	POU class 2 homeobox associating factor 1
*USP43*	−4.12	Ubiquitin-specific peptidase 43	*GLOD5*	7.29	Glyoxalase domain containing 5
*SYNPO2L*	−4.07	Synaptopodin 2 like	*CXCL13L2*	7.25	C-X-C motif chemokine ligand 13-like 2
*SLC10A4*	−4.06	Solute carrier family 10 member 4	*GZMA*	7.17	Granzyme A
*INSM2*	−3.93	INSM transcriptional repressor 2	*AICDA*	7.11	Activation-induced cytidine deaminase
*GPRC5C*	−3.86	G protein-coupled receptor class C group 5 member C	*ART1*	7.10	ADP-ribosyltransferase 1
*PLEKHD1*	−3.68	Pleckstrin homology and coiled-coil domain containing D1	**Highly Downregulated DEGs at 12 wpi**		
*KCNK3*	−3.61	Potassium two-pore domain channel subfamily K member 3	**Gene**	**log_2_FC**	**Description (as Provided on Ensembl)**
*OTOS*	−3.52	Otospiralin	*TTR*	−8.30	Transthyretin
*DMP1*	−3.50	Dentin-matrix acidic phosphoprotein 1	*DMRT2*	−5.24	Doublesex and mab-3-related transcription factor 2
*CARNS1*	−3.46	Carnosine synthase 1	*PPP1R17*	−2.86	Protein phosphatase 1 regulatory subunit 17
*NOS1*	−3.41	Nitric oxide synthase 1	*TLL2*	−2.60	Tolloid like 2
*SHISA6*	−3.30	Shisa family member 6	*BAIAP2L2*	−2.59	BAR/IMD domain containing adaptor protein 2 like 2
			*OvoDA1*	−2.47	Ovodefensin A1
			*KCNH8*	−2.29	Potassium voltage-gated channel subfamily H member 8
			*NPFFR2*	−2.23	Neuropeptide FF receptor 2
			*KY*	−2.18	Kyphoscoliosis peptidase
			*KCNK3*	−2.04	Potassium two-pore domain channel subfamily K member 3

**Table 5:** Total highly differentially expressed novel long non-coding RNAs (lncRNAs) in the brains of ABBV-1 infected Muscovy ducks and chickens.

Species, Time Point	Highly Differentially Expressed Novel lncRNAs (Absolute log_2_FC > 2)		
	Total lncRNAs	Upregulated	Downregulated
Ducks, 4 wpi	95	87	8
Ducks, 12 wpi	22	22	0
Chickens, 4 wpi	13	2	11
Chickens, 12 wpi	123	119	4

**Table 6:** Statistically significant enrichment of KEGG pathways in the brains of ABBV-1 infected Muscovy ducks and chickens.

Pathway Name	Pathway ID	Groups and *p*-Value (adj)		
		Ducks at 4 wpi	Ducks at 12 wpi	Chickens at 12 wpi
Cytokine-cytokine receptor interaction	KEGG:04060	1.597 × 10^−26^	4.112 × 10^−4^	9.672 × 10^−29^
Toll-like receptor signaling pathway	KEGG:04620	9.830 × 10^−11^	5.009 × 10^−3^	2.496 × 10^−9^
Herpes simplex virus 1 infection	KEGG:05168	1.182 × 10^−9^	1.439 × 10^−8^	1.807 × 10^−9^
Cell adhesion molecules	KEGG:04514	6.796 × 10^−9^	6.256 × 10^−10^	4.410 × 10^−15^
Influenza A	KEGG:05164	7.120 × 10^−8^	1.386 × 10^−3^	1.501 × 10^−8^
NOD-like receptor signaling pathway	KEGG:04621	2.323 × 10^−6^	7.947 × 10^−4^	2.031 × 10^−3^
Phagosome	KEGG:04145	7.605 × 10^−5^	7.501 × 10^−3^	1.213 × 10^−7^
Cytosolic DNA-sensing pathway	KEGG:04623	5.904 × 10^−6^	1.852 × 10^−3^	
Intestinal immune network for IgA production	KEGG:04672	4.814 × 10^−8^		6.503 × 10^−22^
Necroptosis	KEGG:04217	7.425 × 10^−4^		
RIG-I-like receptor signaling pathway	KEGG:04622	1.171 × 10^−5^		
C-type lectin receptor signaling pathway	KEGG:04625			1.520 × 10^−2^

No KEGG pathways were significantly enriched for chickens at 4 wpi. Empty cells indicate no enrichment of these pathways. Data were generated using g:Profiler.

## Data Availability

Not applicable.

## Supplementary Materials

The following supporting information can be downloaded at: https://www.mdpi.com/article/10.3390/v14102211/s1. Table S1: Summary of individual-bird ABBV-1 RNA copy-number and histology-score data in brains of ducks and chickens; Table S2: Summary of sequencing analysis in infected and control Muscovy ducks; Table S3: Summary of sequencing analysis in infected and control chickens; Table S4: The 20 most highly up- and downregulated novel differentially expressed genes (DEG) in Muscovy ducks; Table S5: The 20 most highly up- and downregulated novel differentially expressed genes (DEG) in chickens; Table S6: The 20 most highly up- and downregulated novel long non-coding RNAs (lncRNAs) in ducks and chickens.

## References

[1] Rubbenstroth D., Briese T., Dürrwald R., Horie M., Hyndman T.H., Kuhn J.H., Nowotny N., Payne S., Stenglein M.D., Tomonaga K. (2021). ICTV Virus Taxonomy Profile: Bornaviridae. J. Gen. Virol..

[2] Nobach D., Müller J., Tappe D., Herden C. (2020). Update on immunopathology of bornavirus infections in humans and animals. Adv. Virus Res..

[3] Tizard I., Ball J., Stoica G., Payne S. (2016). The pathogenesis of bornaviral diseases in mammals. Anim. Health Res. Rev..

[4] Richt J.A., Grabner A., Herzog S. (2000). Borna disease in horses. Vet. Clin. N. Am. Equine Pract..

[5] Delnatte P., Ojkic D., DeLay J., Campbell D., Crawshaw G., Smith D.A. (2013). Pathology and diagnosis of avian bornavirus infection in wild Canada geese (*Branta canadensis*), trumpeter swans (*Cygnus buccinator*) and mute swans (*Cygnus olor*) in Canada: A retrospective study. Avian Pathol..

[6] Delnatte P., Nagy É., Ojkic D., Leishman D., Crawshaw G., Elias K., Smith D.A. (2014). Avian bornavirus in free-ranging waterfowl: Prevalence of antibodies and cloacal shedding of viral RNA. J. Wildl. Dis..

[7] Murray M., Guo J., Tizard I., Jennings S., Shivaprasad H.L., Payne S., Ellis J.C., Van Wettere A.J., O’Brien K.M. (2017). Aquatic Bird Bornavirus-Associated Disease in Free-Living Canada Geese (*Branta canadensis*) in the Northeastern USA. J. Wildl. Dis..

[8] Thomsen A.F., Nielsen J.B., Hjulsager C.K., Chriél M., Smith D.A., Bertelsen M.F. (2015). Aquatic Bird Bornavirus 1 in Wild Geese, Denmark. Emerg. Infect. Dis..

[9] Świętoń E., Dziadek K., Śmietanka K. (2022). Avian Bornaviruses in Wild Aquatic Birds of the Anseriformes Order in Poland. Pathogens.

[10] Rubbenstroth D., Schmidt V., Rinder M., Legler M., Twietmeyer S., Schwemmer P., Corman V.M. (2016). Phylogenetic Analysis Supports Horizontal Transmission as a Driving Force of the Spread of Avian Bornaviruses. PLoS ONE.

[11] Payne S.L., Delnatte P., Guo J., Heatley J.J., Tizard I., Smith D.A. (2012). Birds and bornaviruses. Anim. Health Res. Rev..

[12] Nielsen A.M.W., Ojkic D., Dutton C.J., Smith D.A. (2018). Aquatic bird bornavirus 1 infection in a captive Emu (*Dromaius novaehollandiae*): Presumed natural transmission from free-ranging wild waterfowl. Avian Pathol..

[13] Guo J., Tizard I., Baroch J., Shivaprasad H.L., Payne S.L. (2015). Avian Bornaviruses in North American Gulls. J. Wildl. Dis..

[14] Rubbenstroth D. (2022). Avian Bornavirus Research-A Comprehensive Review. Viruses.

[15] Iverson M., Leacy A., Pham P.H., Che S., Brouwer E., Nagy E., Lillie B.N., Susta L. (2022). Experimental infection of aquatic bird bornavirus in Muscovy ducks. Sci. Rep..

[16] Iverson M., Leacy A., Pham P.H., Brouwer E., Nagy E., Lillie B.N., Susta L. (2022). Pathogenesis of Aquatic Bird Bornavirus 1 in Domestic Chickens. Res. Sq..

[17] Pham P.H., Leacy A., Deng L., Nagy É., Susta L. (2020). Isolation of Ontario aquatic bird bornavirus 1 and characterization of its replication in immortalized avian cell lines. Virol. J..

[18] Jehle C., Herpfer I., Rauer M., Schwemmle M., Sauder C. (2003). Identification of differentially expressed genes in brains of newborn Borna disease virus-infected rats in the absence of inflammation. Arch. Virol..

[19] Williams B.L., Lipkin W.I. (2006). Endoplasmic reticulum stress and neurodegeneration in rats neonatally infected with borna disease virus. J. Virol..

[20] Lin C.C., Wu Y.J., Heimrich B., Schwemmle M. (2013). Absence of a robust innate immune response in rat neurons facilitates persistent infection of Borna disease virus in neuronal tissue. Cell. Mol. Life Sci..

[21] Liu X., Yang Y., Zhao M., Bode L., Zhang L., Pan J., Lv L., Zhan Y., Liu S., Zhang L. (2014). Proteomics reveal energy metabolism and mitogen-activated protein kinase signal transduction perturbation in human Borna disease virus Hu-H1-infected oligodendroglial cells. Neuroscience.

[22] Suberbielle E., Stella A., Pont F., Monnet C., Mouton E., Lamouroux L., Monsarrat B., Gonzalez-Dunia D. (2008). Proteomic analysis reveals selective impediment of neuronal remodeling upon Borna disease virus infection. J. Virol..

[23] Tang T., Guo Y., Xu X., Zhao L., Shen X., Sun L., Xie P. (2021). BoDV-1 infection induces neuroinflammation by activating the TLR4/MyD88/IRF5 signaling pathway, leading to learning and memory impairment in rats. J. Med. Virol..

[24] Sun L., Guo Y., He P., Xu X., Zhang X., Wang H., Tang T., Zhou W., Xu P., Xie P. (2019). Genome-wide profiling of long noncoding RNA expression patterns and CeRNA analysis in mouse cortical neurons infected with different strains of borna disease virus. Genes Dis..

[25] Bourgey M., Dali R., Eveleigh R., Chen K.C., Letourneau L., Fillon J., Michaud M., Caron M., Sandoval J., Lefebvre F. (2019). GenPipes: An open-source framework for distributed and scalable genomic analyses. Gigascience.

[26] Bolger A.M., Lohse M., Usadel B. (2014). Trimmomatic: A flexible trimmer for Illumina sequence data. Bioinformatics.

[27] Dobin A., Davis C.A., Schlesinger F., Drenkow J., Zaleski C., Jha S., Batut P., Chaisson M., Gingeras T.R. (2013). STAR: Ultrafast universal RNA-seq aligner. Bioinformatics.

[28] Yates A.D., Achuthan P., Akanni W., Allen J., Allen J., Alvarez-Jarreta J., Amode M.R., Armean I.M., Azov A.G., Bennett R. (2020). Ensembl 2020. Nucleic Acids Res..

[29] Anders S., Pyl P.T., Huber W. (2015). HTSeq—A Python framework to work with high-throughput sequencing data. Bioinformatics.

[30] Love M.I., Huber W., Anders S. (2014). Moderated estimation of fold change and dispersion for RNA-seq data with DESeq2. Genome Biol..

[31] Crow M., Lim N., Ballouz S., Pavlidis P., Gillis J. (2019). Predictability of human differential gene expression. Proc. Natl. Acad. Sci. USA.

[32] Sweeney T.E., Haynes W.A., Vallania F., Ioannidis J.P., Khatri P. (2017). Methods to increase reproducibility in differential gene expression via meta-analysis. Nucleic Acids Res..

[33] Benjamini Y., Hochberg Y. (1995). Controlling the False Discovery Rate: A Practical and Powerful Approach to Multiple Testing. J. R Stat. Soc. Ser. B.

[34] Young M.D., Wakefield M.J., Smyth G.K., Oshlack A. (2010). Gene ontology analysis for RNA-seq: Accounting for selection bias. Genome Biol..

[35] Raudvere U., Kolberg L., Kuzmin I., Arak T., Adler P., Peterson H., Vilo J. (2019). g:Profiler: A web server for functional enrichment analysis and conversions of gene lists (2019 update). Nucleic Acids Res..

[36] Sims D., Sudbery I., Ilott N.E., Heger A., Ponting C.P. (2014). Sequencing depth and coverage: Key considerations in genomic analyses. Nat. Rev. Genet..

[37] Conesa A., Madrigal P., Tarazona S., Gomez-Cabrero D., Cervera A., McPherson A., Szcześniak M.W., Gaffney D.J., Elo L.L., Zhang X. (2016). A survey of best practices for RNA-seq data analysis. Genome Biol..

[38] Schurch N.J., Schofield P., Gierliński M., Cole C., Sherstnev A., Singh V., Wrobel N., Gharbi K., Simpson G.G., Owen-Hughes T. (2016). How many biological replicates are needed in an RNA-seq experiment and which differential expression tool should you use?. RNA.

[39] Leacy A., Nagy É., Pham P.H., Susta L. (2020). In Vitro and In Ovo Host Restriction of Aquatic Bird Bornavirus 1 in Different Avian Hosts. Viruses.

[40] Volmer R., Prat C.M.A., Le Masson G., Garenne A., Gonzalez-Dunia D. (2007). Borna disease virus infection impairs synaptic plasticity. J. Virol..

[41] Long L., Zhang X., Chen F., Pan Q., Phiphatwatchara P., Zeng Y., Chen H. (2018). The promising immune checkpoint LAG-3: From tumor microenvironment to cancer immunotherapy. Genes Cancer.

[42] Sobhani N., Tardiel-Cyril D.R., Davtyan A., Generali D., Roudi R., Li Y. (2021). CTLA-4 in Regulatory T Cells for Cancer Immunotherapy. Cancers.

[43] Tappe D., Schmidt-Chanasit J., Rauch J., Allartz P., Herden C. (2019). Immunopathology of Fatal Human Variegated Squirrel Bornavirus 1 Encephalitis, Germany, 2011–2013. Emerg. Infect. Dis..

[44] Hameed S.S., Guo J., Tizard I., Shivaprasad H.L., Payne S. (2018). Studies on immunity and immunopathogenesis of parrot bornaviral disease in cockatiels. Virology.

[45] Leal de Araújo J., Rech R.R., Rodrigues-Hoffmann A., Giaretta P.R., Cirqueira C., Wenceslau R.R., Tizard I., Diaz-Delgado J. (2022). Immunophenotype of the inflammatory response in the central and enteric nervous systems of cockatiels (*Nymphicus hollandicus*) experimentally infected with parrot bornavirus 2. Vet. Pathol..

[46] Huang Y.-H., Feng H.-P., Huang L.-R., Yi K., Rong E.-G., Chen X.-Y., Li J.-W., Wang Z., Zhu P.-Y., Liu X.-J. (2019). Transcriptomic analyses reveal new genes and networks response to H5N1 influenza viruses in duck (*Anas platyrhynchos*). J. Integr. Agric..

[47] Zhang X., Wang W., Zhu W., Dong J., Cheng Y., Yin Z., Shen F. (2019). Mechanisms and Functions of Long Non-Coding RNAs at Multiple Regulatory Levels. Int. J. Mol. Sci..

[48] Yao R.W., Wang Y., Chen L.L. (2019). Cellular functions of long noncoding RNAs. Nat. Cell Biol..

[49] Satpathy A.T., Chang H.Y. (2015). Long noncoding RNA in hematopoiesis and immunity. Immunity.

[50] Walther K., Schulte L.N. (2021). The role of lncRNAs in innate immunity and inflammation. RNA Biol..

[51] Mathy N.W., Chen X.M. (2017). Long non-coding RNAs (lncRNAs) and their transcriptional control of inflammatory responses. J. Biol. Chem..

[52] Buwitt-Beckmann U., Heine H., Wiesmüller K.H., Jung G., Brock R., Akira S., Ulmer A.J. (2006). TLR1- and TLR6-independent recognition of bacterial lipopeptides. J. Biol. Chem..

[53] Takeda K., Akira S. (2005). Toll-like receptors in innate immunity. Int. Immunol..

[54] Park B.S., Lee J.O. (2013). Recognition of lipopolysaccharide pattern by TLR4 complexes. Exp. Mol. Med..

[55] Uchikawa E., Lethier M., Malet H., Brunel J., Gerlier D., Cusack S. (2016). Structural Analysis of dsRNA Binding to Anti-viral Pattern Recognition Receptors LGP2 and MDA5. Mol. Cell.

[56] Satoh T., Kato H., Kumagai Y., Yoneyama M., Sato S., Matsushita K., Tsujimura T., Fujita T., Akira S., Takeuchi O. (2010). LGP2 is a positive regulator of RIG-I- and MDA5-mediated antiviral responses. Proc. Natl. Acad. Sci. USA.

[57] Maringer K., Fernandez-Sesma A. (2014). Message in a bottle: Lessons learned from antagonism of STING signalling during RNA virus infection. Cytokine Growth Factor Rev..

[58] Olejnik J., Hume A.J., Mühlberger E. (2018). Toll-like receptor 4 in acute viral infection: Too much of a good thing. PLoS Pathog..

[59] Miyashita M., Oshiumi H., Matsumoto M., Seya T. (2011). DDX60, a DEXD/H box helicase, is a novel antiviral factor promoting RIG-I-like receptor-mediated signaling. Mol. Cell. Biol..

[60] Gack M.U., Shin Y.C., Joo C.H., Urano T., Liang C., Sun L., Takeuchi O., Akira S., Chen Z., Inoue S. (2007). TRIM25 RING-finger E3 ubiquitin ligase is essential for RIG-I-mediated antiviral activity. Nature.

[61] Lian H., Zang R., Wei J., Ye W., Hu M.M., Chen Y.-D., Zhang X.N., Guo Y., Lei C.Q., Yang Q. (2018). The Zinc-Finger Protein ZCCHC3 Binds RNA and Facilitates Viral RNA Sensing and Activation of the RIG-I-like Receptors. Immunity.

[62] Zhou Z., Jia X., Xue Q., Dou Z., Ma Y., Zhao Z., Jiang Z., He B., Jin Q., Wang J. (2014). TRIM14 is a mitochondrial adaptor that facilitates retinoic acid-inducible gene-I-like receptor-mediated innate immune response. Proc. Natl. Acad. Sci. USA.

[63] Ning S., Pagano J.S., Barber G.N. (2011). IRF7: Activation, regulation, modification and function. Genes Immun..

[64] Au-Yeung N., Mandhana R., Horvath C.M. (2013). Transcriptional regulation by STAT1 and STAT2 in the interferon JAK-STAT pathway. JAK-STAT.

[65] Schneider W.M., Chevillotte M.D., Rice C.M. (2014). Interferon-stimulated genes: A complex web of host defenses. Annu. Rev. Immunol..

[66] Staeheli P., Sentandreu M., Pagenstecher A., Hausmann J. (2001). Alpha/beta interferon promotes transcription and inhibits replication of borna disease virus in persistently infected cells. J. Virol..

[67] Nailwal H., Chan F.K.M. (2019). Necroptosis in anti-viral inflammation. Cell Death Differ..

[68] Deschl U., Stitz L., Herzog S., Frese K., Rott R. (1990). Determination of immune cells and expression of major histocompatibility complex class II antigen in encephalitic lesions of experimental Borna disease. Acta Neuropathol..

[69] Bilzer T., Stitz L. (1994). Immune-mediated brain atrophy. CD8+ T cells contribute to tissue destruction during borna disease. J. Immunol..

[70] Planz O., Bilzer T., Stitz L. (1995). Immunopathogenic role of T-cell subsets in Borna disease virus-induced progressive encephalitis. J. Virol..

[71] Planz O., Stitz L. (1999). Borna disease virus nucleoprotein (p40) is a major target for CD8^+^-T-cell-mediated immune response. J. Virol..

[72] Chen D., Yu J., Zhang L. (2016). Necroptosis: An alternative cell death program defending against cancer. Biochim. Biophys. Acta-Rev. Cancer.

