# Supplementary material for: Transcriptome Analysis of Duck and Chicken Brains Infected with Aquatic Bird Bornavirus-1 (ABBV-1)
Source: Viruses. 2022 Oct 8;14(10):2211. doi: 10.3390/v14102211 (PMC9611670; doi:10.3390/v14102211)
Supplement: Supplementary file 1 [file viruses-14-02211-s001.zip › viruses-1939363-supplementary.pdf]

Supplementary Materials

Table S1. Summary of individual bird ABBV-1 RNA copy number and histology score data in brains of ducks and chickens.

Bird Identifier[1]	Weeks Post-infection	ABBV-1 RNA Copy Number[2]	Histology Score[3]
D501	4	7.09	2.0
D508	4	7.21	3.0
D509	4	5.98	4.0
D513	4	7.18	3.0
D522	4	7.06	3.0
D524	4	6.57	3.0
D544	4	7.01	3.5
D503	12	7.14	2.0
D504	12	7.33	1.0
D526	12	7.09	3.0
D506	12	7.12	2.0
D507	12	7.14	2.0
D521	12	7.13	2.0
D525	12	7.10	1.5
C114	4	3.51	0
C117	4	6.32	0
C125	4	3.72	0
C131	4	5.62	0
C133	4	3.69	0
C134	4	5.51	0
C139	4	6.40	0
C104	12	7.18	3.5
C105	12	5.83	2.0
C106	12	6.05	5.5
C111	12	7.03	4.5
C124	12	6.22	4.3
C127	12	7.08	3.7
C140	12	6.52	3.8

[1] Each bird was identified separately using a code. For ducks, the identifier began with “D” followed by a number in the 500 range for infected birds and in the 800 range for control birds. For chickens, the identifier began with “C” followed by a number in the 100 range for infected birds and in the 400 range for control birds. Only birds exposed to ABBV-1 are shown since control birds tested negative for ABBV-1 by RT-qPCR and showed no inflammation.

[2] Log10 virus RNA copies / 150 ng of tissue RNA.

[3] Semi-quantitative severity of microscopic inflammation (0-6).

Data adapted from Iverson et al. [15] and Iverson et al. [16].

Table S2. Summary of sequencing analysis in infected and control ducks

Sample[1]	Raw Reads	Surviving Reads	Surviving Reads (%)	Aligned Reads	Reads Aligned (%)	Mean Coverage Per Base	Genes
D501	92,052,122	92,022,688	99.97	67,491,420	73.34	47.41	9942
D503	105,799,486	105,775,660	99.98	80,185,031	75.81	54.63	9871
D504	128,130,980	128,089,986	99.97	93,038,833	72.64	59.32	10080
D506	105,351,130	105,322,586	99.97	78,703,384	74.73	53.31	9883
D507	110,641,640	110,612,624	99.97	84,187,758	76.11	55.99	9912
D508	98,497,204	98,477,728	99.98	77,071,051	78.26	53.91	10004
D509	91,840,532	91,824,644	99.98	71,471,183	77.83	50.21	10006
D513	94,933,588	94,907,252	99.97	70,157,457	73.92	48.39	9949
D521	96,605,420	96,575,988	99.97	72,360,454	74.93	49.80	9833
D522	122,038,990	122,011,076	99.98	93,175,575	76.37	64.47	9912
D524	112,061,552	111,999,544	99.94	86,010,505	76.80	58.20	9934
D525	98,021,016	97,991,816	99.97	73,774,097	75.29	51.47	9779
D526	95,660,234	95,629,156	99.97	72,356,902	75.66	47.67	9792
D544	100,006,296	99,978,392	99.97	76,319,266	76.34	52.97	9912
D801	99,744,778	99,722,322	99.98	77,151,071	77.37	56.43	9821
D804	136,268,072	136,235,632	99.98	109,746,995	80.56	80.03	9896
D809	86,649,730	86,629,104	99.98	68,217,240	78.75	49.49	9787
D810	94,610,958	94,592,716	99.98	72,749,313	76.91	51.79	9758
D811	89,476,392	89,453,330	99.97	69,747,689	77.97	49.27	9810
D814	66,851,188	66,834,424	99.97	53,167,653	79.55	38.44	9703
D816	114,876,394	114,848,348	99.98	87,343,231	76.05	57.38	10017
D819	90,986,520	90,962,266	99.97	69,149,176	76.02	50.73	9758
D820	111,699,116	111,670,330	99.97	85,686,583	76.73	59.87	9894
D821	88,513,744	88,494,472	99.98	69,892,846	78.98	48.31	9915
D822	108,677,928	108,652,292	99.98	86,389,094	79.51	60.23	9828
D824	98,564,074	98,540,136	99.98	76,167,795	77.30	54.20	9802
D829	92,476,548	92,453,962	99.98	72,058,800	77.94	54.28	9768
D836	96,384,640	96,360,398	99.97	75,423,029	78.27	54.22	9857

[1] Each bird was identified separately using a code. For ducks, the identifier began with “D” followed by a number in the 500 range for infected birds and in the 800 range for control birds.

Table S3. Summary of sequencing analysis in infected and control chickens

Sample[1]	Raw Reads	Surviving Reads	Surviving Reads (%)	Aligned Reads	Reads Aligned (%)	Mean Coverage Per Base	Genes
C104	170,553,534	170,491,518	99.96	157,453,838	92.32	98.56	11,961
C105	82,253,152	82,224,678	99.97	76,614,839	93.15	54.89	11,830
C106	85,697,124	85,663,430	99.96	79,944,905	93.29	49.75	11,793
C111	91,366,276	91,339,968	99.97	85,329,583	93.39	55.86	11,793
C114	109,319,382	109,301,638	99.98	105,541,702	96.54	72.98	11,724
C117	108,024,342	107,992,970	99.97	103,849,133	96.13	74.22	11,743
C124	101,526,562	101,485,028	99.96	93,362,631	91.96	58.00	11,836
C125	109,485,062	109,447,364	99.97	105,056,575	95.96	73.24	11,854
C127	119,335,264	119,287,442	99.96	109,820,103	92.03	68.26	11,964
C131	110,499,004	110,467,034	99.97	106,071,134	95.99	73.34	11,807
C133	95,493,706	95,472,606	99.98	92,251,102	96.60	65.39	11,886
C134	136,947,300	136,917,580	99.98	131,480,101	96.01	93.57	11,856
C139	83,791,650	83,767,934	99.97	80,304,791	95.84	57.70	11,818
C140	96,985,440	96,935,976	99.95	88,501,009	91.25	53.80	11,836
C403	83,833,816	83,813,664	99.98	79,375,323	94.68	58.74	11,851
C404	102,120,914	102,088,234	99.97	96,229,198	94.23	67.45	11,901
C407	85,518,226	85,490,602	99.97	80,666,304	94.33	59.62	11,802
C409	143,772,338	143,735,444	99.97	137,577,953	95.69	101.57	11,990
C417	97,030,904	97,006,860	99.98	91,438,115	94.24	67.64	11,837
C419	104,177,232	104,147,948	99.97	98,349,251	94.41	66.87	11,697
C425	99,279,684	99,253,868	99.97	94,520,756	95.21	65.91	11,814
C427	95,074,400	95,046,088	99.97	89,810,802	94.46	63.48	11,823
C428	100,897,280	100,868,378	99.97	95,772,064	94.92	67.25	11,818
C429	82,578,244	82,556,586	99.97	78,491,869	95.05	56.14	11,735
C432	102,381,214	102,352,790	99.97	97,425,566	95.16	69.48	11,771
C436	112,510,720	112,488,264	99.98	107,953,957	95.95	75.70	11,790
C439	84,298,534	84,266,556	99.96	79,042,602	93.77	54.61	11,712
C442	104,205,752	104,169,724	99.97	97,950,061	94.00	74.52	11,832

[1] Each bird was identified separately using a code. For chickens, the identifier began with "C" followed by a number in the 100 range for infected birds and in the 400 range for control birds.

Table S4. Top 20 highly up- and downregulated novel differentially expressed genes (DEG) in ducks

Duck 4 wpi highly upregulated DEG		
**Gene**	**Log₂FC[1]**	**Protein Name (as provided in UniProt)**
ENSAPLG00000026791	12.48	Ig-like domain-containing protein
ENSAPLG00000016552	11.84	Ig-like domain-containing protein
ENSAPLG00000028125	11.71	Ig-like domain-containing protein
ENSAPLG00000024383	11.64	Uncharacterized protein
ENSAPLG00000021063	11.56	Ig-like domain-containing protein
ENSAPLG00000019893	11.38	Ig-like domain-containing protein
ENSAPLG00000031161	11.36	Ig-like domain-containing protein
ENSAPLG00000016821	11.16	Ig-like domain-containing protein
ENSAPLG00000018830	11.15	Ig-like domain-containing protein
ENSAPLG00000024467	10.86	Ig-like domain-containing protein
ENSAPLG00000021993	10.72	Ig-like domain-containing protein
ENSAPLG00000016883	10.64	Uncharacterized protein
ENSAPLG00000021802	10.58	Uncharacterized protein
ENSAPLG00000025736	10.50	Ig-like domain-containing protein
ENSAPLG00000030169	10.43	Ig-like domain-containing protein
ENSAPLG00000024563	10.37	Ig-like domain-containing protein
ENSAPLG00000029471	10.25	Ig-like domain-containing protein
ENSAPLG00000027840	10.22	Ig-like domain-containing protein
ENSAPLG00000025655	10.13	SCY domain-containing protein
ENSAPLG00000025965	9.94	Uncharacterized protein

Duck 4 wpi highly downregulated DEG		
**Gene**	**Log₂FC**	**Description (as provided in Ensembl[2])**
ENSAPLG00000019061	-4.56	Long non-coding RNA
ENSAPLG00000029485	-3.89	Long non-coding RNA
ENSAPLG00000028039	-2.79	Long non-coding RNA
ENSAPLG00000018213	-2.39	Long non-coding RNA
ENSAPLG00000021011	-2.26	Long non-coding RNA
ENSAPLG00000020436	-2.18	Long non-coding RNA
ENSAPLG00000022296	-2.15	Long non-coding RNA
ENSAPLG00000028968	-2.13	Long non-coding RNA

Duck 12 wpi highly upregulated DEG		
**Gene**	**Log₂FC**	**Protein Name (as provided in UniProt)**
ENSAPLG00000031161	10.67	Ig-like domain-containing protein
ENSAPLG00000019893	9.82	Ig-like domain-containing protein
ENSAPLG00000026791	9.56	Ig-like domain-containing protein
ENSAPLG00000011142	9.26	Uncharacterized protein
ENSAPLG00000028125	8.83	Ig-like domain-containing protein
ENSAPLG00000016552	8.28	Ig-like domain-containing protein
ENSAPLG00000021802	8.22	Uncharacterized protein
ENSAPLG00000023118	8.14	Ig-like domain-containing protein
ENSAPLG00000025965	8.11	Uncharacterized protein
ENSAPLG00000002226	8.07	Ig-like domain-containing protein
ENSAPLG00000024467	7.63	Ig-like domain-containing protein
ENSAPLG00000024383	7.46	Uncharacterized protein
ENSAPLG00000028723	7.34	Ig-like domain-containing protein
ENSAPLG00000020906	7.21	Ig-like domain-containing protein
ENSAPLG00000031023	7.10	SCY domain-containing protein
ENSAPLG00000021063	7.08	Ig-like domain-containing protein

ENSAPLG00000001610	6.80	Uncharacterized protein
ENSAPLG00000016883	6.75	Uncharacterized protein
ENSAPLG00000027840	6.50	Ig-like domain-containing protein
ENSAPLG00000020955	6.49	Ig-like domain-containing protein

[1] $Log_2FC = Log_2$ Fold Change
[2] Ensembl release 106: Apr 2022

Table S5. Top 20 highly up- and downregulated novel differentially expressed genes (DEG) in chickens

**Chicken 4 wpi highly upregulated DEG**		
**Gene**	**Log_2FC[1]**	**Description (Ensembl[2]) or Protein Name (UniProt)**
ENSGALG00000052161	2.64	Long non-coding RNA
ENSGALG00000009479	2.04	Uncharacterized protein
ENSGALG00000052516	2.02	Long non-coding RNA

**Chicken 4 wpi highly downregulated DEG**		
**Gene**	**Log_2FC**	**Description (Ensembl) or Protein Name (UniProt)**
ENSGALG00000053701	-4.55	G_PROTEIN_RECEP_F3_4 domain-containing protein
ENSGALG00000053055	-3.56	B30.2/SPRY domain-containing protein
ENSGALG00000048771	-3.52	Uncharacterized protein
ENSGALG00000049586	-3.47	Long non-coding RNA
ENSGALG00000029331	-3.39	Long non-coding RNA
ENSGALG00000052021	-3.34	Long non-coding RNA
ENSGALG00000032417	-3.26	DNA helicase
ENSGALG00000054795	-3.18	TED_complement domain-containing protein
ENSGALG00000038532	-3.17	Uncharacterized protein
ENSGALG00000048945	-3.13	Long non-coding RNA
ENSGALG00000011813	-3.11	EGF-like domain-containing protein
ENSGALG00000051290	-3.06	Uncharacterized protein
ENSGALG00000047027	-2.00	ABC1 domain-containing protein
ENSGALG00000053449	-2.91	Uncharacterized protein
ENSGALG00000054594	-2.91	Long non-coding RNA
ENSGALG00000007740	-2.82	Wiskott-Aldrich syndrome protein family member
ENSGALG00000043906	-2.79	chicken D-serine dehydratase
ENSGALG00000051469	-2.69	Uncharacterized protein
ENSGALG00000045762	-2.68	Uncharacterized protein
ENSGALG00000030587	-2.68	PH domain-containing protein

**Chicken 12 wpi highly upregulated DEG**		
**Gene**	**Log_2FC**	**Protein Name (as provided in UniProt)**
ENSGALG00000050545	14.34	Ig-like domain-containing protein
ENSGALG00000048383	14.27	Ig-like domain-containing protein
ENSGALG00000046719	13.23	Ig-like domain-containing protein
ENSGALG00000047440	13.15	Ig-like domain-containing protein
ENSGALG00000047866	12.87	Ig-like domain-containing protein
ENSGALG00000053057	12.59	Ig-like domain-containing protein
ENSGALG00000050515	12.47	Ig-like domain-containing protein
ENSGALG00000049267	11.91	Ig-like domain-containing protein
ENSGALG00000052142	11.85	Ig-like domain-containing protein
ENSGALG00000015662	11.83	Lipase domain-containing protein
ENSGALG00000050477	11.51	Ig-like domain-containing protein
ENSGALG00000051617	11.38	Ig-like domain-containing protein
ENSGALG00000050023	11.38	Ig-like domain-containing protein

ENSGALG00000052651	11.34	Ig-like domain-containing protein
ENSGALG00000052542	11.27	Ig-like domain-containing protein
ENSGALG00000054874	11.26	Ig-like domain-containing protein
ENSGALG00000049450	11.22	Immunoglobulin lambda like polypeptide 1
ENSGALG00000047960	11.03	Ig-like domain-containing protein
ENSGALG00000054437	10.97	Ig-like domain-containing protein
ENSGALG00000055104	10.95	Ig-like domain-containing protein

**Chicken 12 wpi highly downregulated DEG**		
**Gene**	**Log$_2$FC**	**Description (Ensembl) or Protein Name (UniProt)**
ENSGALG00000053641	-3.36	Uncharacterized protein
ENSGALG00000047107	-3.08	Long non-coding RNA
ENSGALG00000045001	-2.85	Long non-coding RNA
ENSGALG00000043906	-2.74	chicken D-serine dehydratase
ENSGALG00000052075	-2.63	Uncharacterized protein
ENSGALG00000029168	-2.37	Collectrin domain-containing protein
ENSGALG00000046845	-2.18	Long non-coding RNA
ENSGALG00000051605	-2.09	Long non-coding RNA

[1] Log$_2$FC = Log$_2$ Fold Change

[2] Ensembl release 106: Apr 2022

Table S6. Top 20 highly up- and downregulated novel long non-coding RNAs (lncRNAs) in ducks and chickens

**Duck 4 wpi highly upregulated lncRNAs**	
**Gene**	**Log$_2$FC[1]**
ENSAPLG00000023351	7.33
ENSAPLG00000027245	7.02
ENSAPLG00000030029	7.02
ENSAPLG00000023929	6.48
ENSAPLG00000028494	6.10
ENSAPLG00000012096	6.00
ENSAPLG00000024074	5.96
ENSAPLG00000018036	5.92
ENSAPLG00000021010	5.57
ENSAPLG00000016648	5.54
ENSAPLG00000023964	5.32
ENSAPLG00000017123	5.30
ENSAPLG00000023775	5.28
ENSAPLG00000017122	5.26
ENSAPLG00000022141	5.19
ENSAPLG00000028601	5.06
ENSAPLG00000026563	5.03
ENSAPLG00000018634	4.92
ENSAPLG00000020790	4.90
ENSAPLG00000026448	4.82

**Duck 4 wpi highly downregulated lncRNAs**	
**Gene**	**Log$_2$FC**
ENSAPLG00000019061	-4.56
ENSAPLG00000029485	-3.89
ENSAPLG00000028039	-2.79
ENSAPLG00000018213	-2.39

**Chicken 4 wpi highly upregulated lncRNAs**	
**Gene**	**Log$_2$FC**
ENSGALG00000052161	2.64
ENSGALG00000052516	2.02

**Chicken 4 wpi highly downregulated lncRNAs**	
**Gene**	**Log$_2$FC**
ENSGALG00000049586	-3.47
ENSGALG00000029331	-3.39
ENSGALG00000052021	-3.34
ENSGALG00000048945	-3.13
ENSGALG00000054594	-2.91
ENSGALG00000051742	-2.62
ENSGALG00000043657	-2.58
ENSGALG00000043925	-2.29
ENSGALG00000048184	-2.28
ENSGALG00000050200	-2.17
ENSGALG00000053368	-2.12

**Chicken 12 wpi highly upregulated lncRNAs**	
**Gene**	**Log$_2$FC**
ENSGALG00000052959	7.95
ENSGALG00000047851	7.45
ENSGALG00000051998	7.40
ENSGALG00000051039	6.87
ENSGALG00000049423	6.82
ENSGALG00000053060	6.66
ENSGALG00000004167	6.54
ENSGALG00000019325	6.18

ENSAPLG00000021011	-2.26	ENSGALG00000054932	6.16
ENSAPLG00000020436	-2.18	ENSGALG00000037363	6.15
ENSAPLG00000022296	-2.15	ENSGALG00000053738	6.12
ENSAPLG00000028968	-2.13	ENSGALG00000046879	6.11
		ENSGALG00000047054	6.10
**Duck 12 wpi highly upregulated lncRNAs**		ENSGALG00000002102	6.10
**Gene**	**Log_2FC**	ENSGALG00000047087	5.62
ENSAPLG00000021010	5.65	ENSGALG00000053080	5.41
ENSAPLG00000020790	5.29	ENSGALG00000041611	5.33
ENSAPLG00000023929	4.85	ENSGALG00000033094	5.24
ENSAPLG00000012096	4.84	ENSGALG00000052451	4.96
ENSAPLG00000029118	4.61	ENSGALG00000051565	4.92
ENSAPLG00000023351	4.24		
ENSAPLG00000025609	3.58	**Chicken 12 wpi highly downregulated lncRNAs**	
ENSAPLG00000023152	3.28	**Gene**	**Log_2FC**
ENSAPLG00000021565	3.22	ENSGALG00000047107	-3.08
ENSAPLG00000029930	3.01	ENSGALG00000045001	-2.85
ENSAPLG00000017062	2.85	ENSGALG00000046845	-2.18
ENSAPLG00000022020	2.85	ENSGALG00000051605	-2.09
ENSAPLG00000026217	2.68		
ENSAPLG00000022079	2.52		
ENSAPLG00000021959	2.40		
ENSAPLG00000020060	2.24		
ENSAPLG00000020403	2.22		
ENSAPLG00000021446	2.21		
ENSAPLG00000019869	2.18		
ENSAPLG00000021854	2.17		

[1] Log_2FC = Log_2 Fold Change

Not all groups had 20 highly differentially expressed lncRNAs. Ducks at 12 wpi had zero highly downregulated lncRNAs. Ensembl release 106: Apr 2022.
